# Differential involvement of cAMP/PKA-, PLC/PKC- and Ca^2+^/calmodulin-dependent pathways in GnRH-induced prolactin secretion and gene expression in grass carp pituitary cells

**DOI:** 10.3389/fendo.2024.1399274

**Published:** 2024-06-04

**Authors:** Wensheng Li, Cheng Ye, Mulan He, Wendy K. W. Ko, Christopher H. K. Cheng, Ying Wai Chan, Anderson O. L. Wong

**Affiliations:** ^1^ School of Biological Sciences, The University of Hong Kong, Hong Kong, Hong Kong SAR, China; ^2^ School of Life Sciences, Sun Yat-Sen University, Guangzhou, China; ^3^ School of Biomedical Sciences, Chinese University of Hong Kong, Hong Kong, Hong Kong SAR, China

**Keywords:** GnRH, prolactin, hormone secretion, gene expression, signal transduction, pituitary cells, grass carp

## Abstract

Gonadotropin-releasing hormone (GnRH) is a key stimulator for gonadotropin secretion in the pituitary and its pivotal role in reproduction is well conserved in vertebrates. In fish models, GnRH can also induce prolactin (PRL) release, but little is known for the corresponding effect on PRL gene expression as well as the post-receptor signalling involved. Using grass carp as a model, the functional role of GnRH and its underlying signal transduction for PRL regulation were examined at the pituitary level. Using laser capture microdissection coupled with RT-PCR, GnRH receptor expression could be located in carp lactotrophs. In primary cell culture prepared from grass carp pituitaries, the native forms of GnRH, GnRH2 and GnRH3, as well as the GnRH agonist [D-Arg^6^, Pro^9^, NEt]-sGnRH were all effective in elevating PRL secretion, PRL mRNA level, PRL cell content and total production. In pituitary cells prepared from the rostral pars distalis, the region in the carp pituitary enriched with lactotrophs, GnRH not only increased cAMP synthesis with parallel CREB phosphorylation and nuclear translocation but also induced a rapid rise in cytosolic Ca^2+^ by Ca^2+^ influx via L-type voltage-sensitive Ca^2+^ channel (VSCC) with subsequent CaM expression and NFAT_2_ dephosphorylation. In carp pituitary cells prepared from whole pituitaries, GnRH-induced PRL secretion was reduced/negated by inhibiting cAMP/PKA, PLC/PKC and Ca^2+^/CaM/CaMK-II pathways but not the signalling events via IP_3_ and CaN/NFAT. The corresponding effect on PRL mRNA expression, however, was blocked by inhibiting cAMP/PKA/CREB/CBP and Ca^2+^/CaM/CaN/NFAT_2_ signalling but not PLC/IP_3_/PKC pathway. At the pituitary cell level, activation of cAMP/PKA pathway could also induce CaM expression and Ca^2+^ influx via VSCC with parallel rises in PRL release and gene expression in a Ca^2+^/CaM-dependent manner. These findings, as a whole, suggest that the cAMP/PKA-, PLC/PKC- and Ca^2+^/CaM-dependent cascades are differentially involved in GnRH-induced PRL secretion and PRL transcript expression in carp lactotrophs. During the process, a functional crosstalk between the cAMP/PKA- and Ca^2+^/CaM-dependent pathways may occur with PRL release linked with CaMK-II and PKC activation and PRL gene transcription caused by nuclear action of CREB/CBP and CaN/NFAT_2_ signalling.

## 1 Introduction

Gonadotropin-releasing hormone (GnRH), a member of the GnRH/AKH/CRZ superfamily, is an ancient hormone evolved prior to the divergence of protostomes and deuterostomes (1). In vertebrate species, three forms of GnRH have been identified, namely GnRH1, GnRH2 and GnRH3, and believed to be arose from whole genome duplication occurred in ancestral vertebrates (2). Given that multiple forms of GnRH structurally comparable to vertebrate GnRHs can be identified in tunicates, the possibility of GnRH gene duplication occurred during the evolution of protochordate cannot be excluded (3). The three isoforms of GnRH in vertebrate species are highly conserved in a.a. sequence, expressed in a species-specific manner in neurons within different brain areas, and proposed to have overlapping and yet distinct functions caused by subfunctionalization of the respective paralogs (4). In mammals, GnRH1 is the major form and GnRH3 was lost during evolution, whereas GnRH2 can be found in some species (e.g., human and monkey) but not the others (e.g., rodents). In tetrapods like the amphibians, reptiles and birds, GnRH3 was also lost with only GnRH1 and GnRH2 expressed. In fish, different combinations of the three isoforms can be noted, e.g., GnRH1 and GnRH2 in catfish and eel, GnRH2 and GnRH3 in salmon, goldfish and zebrafish, and three forms of GnRH in sea bass, medaka and tilapia (3, 4). At present, our understanding on the neuro-anatomical distribution and differential functions of different GnRH isoforms is mainly based on the studies in fish models (5, 6). In fish with three forms of GnRH (e.g., sea bass), GnRH1 neurons are located in the preoptic area within the brain with notable innervation in the pituitary while GnRH3 neurons are scattering along the terminal nerve, ventral telencephalon and preoptic area with a low level of pituitary innervation. GnRH2 neurons, however, are confined within the midbrain tegmentum with little/no fibres innervating the pituitary (7). Similar to fish models, localization of GnRH1 neurons in the preoptic area (& GnRH2 neurons in the midbrain area of some species) is well-conserved in mammals (8, 9).

In general, the GnRH1 neurons in the preoptico-hypothalamic tract represent the major source of GnRH for gonadotropin regulation at the pituitary level whereas GnRH2 and GnRH3 from other brain areas are commonly accepted to be neurotransmitters/neuromodulators with other functions related to reproduction (4, 9). For examples, GnRH2 from the midbrain area is involved in sexual behaviours, appetite control, steroidogenesis, and implantation/placental functions, and probably also coordinates the interaction between nutritional status and sexual activities (10). Meanwhile, GnRH3 originated from the terminal nerve ganglia has been proposed to play a role in relaying the sensory input from the olfactory bulbs (via olfactory tract) and visual system (from optic tectum) with reproductive functions (4). In medaka, GnRH1 neurons with pituitary innervation exhibit episodic bursting of action potentials related to the reproduction cycle, which is different from the regular pattern of action potentials in GnRH2 and 3 neurons with intrinsic pacemaker activity (11). In the same model, the frequency of action potentials in GnRH1 neurons can be elevated with LH surge (12) and GnRH1 knockout inhibits ovulation and spawning activity in female fish (13). Episodic firing of GnRH1 neurons in the preoptic area has also been reported in tilapia (14), which is consistent with the idea of GnRH pulsatility caused by KNDy neuron network within the hypothalamus leading to episodic release of luteinizing hormone (LH) at the pituitary level (15). In fish models, the functional role of GnRH1 has been replaced by GnRH3 in fish species with only GnRH2 and GnRH3 expression (e.g., the cyprinids and salmonids). In goldfish, a member of the cyprinid family, GnRH3 neurons in the preoptic area have fibres penetrating into the pituitary (16) and GnRH3 is the key mediator for seasonal changes in LH secretion (17) and spawning activity (18). For GnRH2, besides its expression in the midbrain area, it is also expressed in the forebrain (including the preoptic area) as well as in the pituitary (19, 20). In goldfish pituitary cells, interestingly, GnRH2 is more potent than GnRH3 in stimulating LH and follicle-stimulating hormone (FSH) expression (21). GnRH2 fibres (22) and protein signals (23) can also be detected in the pituitary of zebrafish but the source of GnRH2 is still unclear.

The biological actions of GnRH, especially for gonadotropin regulation, are mediated by GnRH receptor (GnRHR) functionally coupled with the PLC/IP_3_/PKC, AC/cAMP/PKA, PLA_2_/AA, PI3K/Akt, MAPK and Ca^2+^-dependent cascades (24, 25) and the post-receptor signalling involved, despite the species-specific variations, is highly conserved from fish to mammals (26, 27). GnRHR is a member of the rhodopsin-like G protein-coupled receptor (GPCR), and its origin can be traced back to the early members of invertebrates (1). In vertebrate species, two types of GnRHR, GnRHR-I and GnRHR-II, can be identified and believed to be the result of a tandem duplication of GnRHR gene in ancestral gnathostome occurred prior to the multiple rounds of genome duplication during vertebrate evolution (28). Subsequent local duplication of GnRHR-II gene followed by additional gene duplication of GnRHR-I and -II along with the whole genome duplication (with 3 rounds in fish and 2 rounds in tetrapods) probably have led to the multiple subtypes of GnRHR-I and -II found in modern-day vertebrates (2, 29). During the course of evolution, GnRHR-I is lost in bony fish and amphibians, while GnRHR-II is lost in most of the mammals (e.g., human and rodents) but retained in some primates (e.g., monkey) (2). Although GHRHR-I and -II share a notable level of sequence homology, the two receptors are structurally and functionally distinct from each other. By comparing with GnRHR-II, the 3^rd^ intracellular loop is relatively shorter and the C-terminal tail typical of GPCR is missing in GnRHR-I (24). In expression studies using GnRHR-II with deletion of the C-terminal tail (30) and GnRHR-I with addition of C-terminal tail from GnRHR-II (31), GnRHR-I, unlike GnRHR-II, was found to be not sensitive to receptor desensitization caused by GnRH and this unique property associated with the lack of C-terminal tail has been recently proven to be essential for normal fertility in the mouse model (32). In general, GnRHR-I is specific for GnRH1 (e.g., mammals) (33) while GnRHR-II (e.g., fish species) is either with a preference for GnRH2 (34) or being promiscuous to the three forms of GnRH (35). Using site-directed mutagenesis and domain swapping in human/monkey GnRHRs, the extracellular loop 2 and 3 together with the residues located close to membrane surface in TMD4 and 7 were found to be the structural determinants for ligand selectivity against different forms of GnRH (36, 37).

In fish models (e.g., tilapia), different subtypes of GnRHR-I and -II are expressed in pituitary cells other than gonadotrophs (38), and GnRH, besides its role as the gonadotropin-releasing factor, can also stimulate growth hormone (GH), prolactin (PRL) and somatolactin (SL) secretion at the pituitary level (27). The PRL response induced by GnRH is particularly interesting as PRL is involved in spawning migration (e.g., in salmon), nesting and brooding (e.g., in birds), parental care (e.g., mouth brooding in fish) and nutrient provision for the young (e.g., lactation in mammals and chick feeding in birds) (for reviews on reproductive functions of PRL, see (39, 40)). Apparently, PRL induction by GnRH may serve as a signal input from the hypothalamo-pituitary axis to trigger the reproductive functions of PRL. Although PRL regulation by GnRH has also been reported in mammals, the information is restricted to PRL release and the results obtained are not consistent, as stimulatory (41–43) and no effect have been documented (44, 45). For stimulatory action, GnRH-induced PRL secretion could be associated with the elevation of IP_3_ (46) and cAMP levels (47, 48) or indirect action via local signals produced by gonadotrophs (49, 50). Except for the limited studies in tilapia for PRL release (51, 52) and in salmon for PRL mRNA expression caused by GnRH (53, 54), little is known for PRL regulation by GnRH in non-mammalian species. Despite a single report in tilapia confirming the involvement of PLC/IP_3_- and Ca^2+^-dependent mechanisms in GnRH-induced PRL secretion in fish model (51), the post-receptor signalling for the effect on PRL gene expression is still unknown. Given that (i) the reproductive functions of PRL are expected to have beneficial effects on induced spawning in commercial fish with GnRH treatment, and (ii) the studies on signal transduction mediating the PRL responses by GnRH are still fragmentary and restricted to PRL release, an *in vitro* study was initiated in carp pituitary cells to examine the pituitary action of GnRH and the post-receptor signalling for GnRH-induced PRL release and gene expression. In our study, grass carp was used as the animal model as it is a representative of the cyprinids with high market value in Asian countries (~5.8 million tonnes/year and accounts for 12% of global finfish culture, data from 2022 FAO Report for World Fisheries and Aquaculture). Based on our promoter analysis of grass carp PRL gene, one CRE site (with core sequence “CGTCA”), one AP-1 site (with core sequence “TCAGTCA”) and three NFAT binding sites (with core sequence “GGAAA”) can be identified in the proximal promoter upstream of the TATA box (**Supplementary Figure S1**), implying that PRL expression in carp pituitary is under the control of cAMP/PKA-, PLC/PKC- and/or Ca^2+^-dependent signalling. Using a pharmacological approach with parallel monitoring of second messengers and transcription factors linked with these signalling pathways, we have demonstrated for the first time that the cAMP/PKA, PLC/PKC and Ca^2+^/Calmodulin (CaM)-dependent cascades together with the crosstalk of cAMP/PKA pathway with CaM expression and Ca^2+^ entry via voltage-sensitive Ca^2+^ channels (VSCC) are differentially involved in PRL release and gene expression caused by GnRH via a specific subtype of GnRHR, namely GnRHR_4_, expressed in lactotrophs in the carp pituitary.

## 2 Materials and methods

### 2.1 Animals and test substances

One-year-old grass carp (*Ctenopharyngodon idellus*) with body weight of ~1.5 kg were purchased from local wholesale markets and acclimated for 5 days in well-aerated 200 L water tanks under a 12-hr light:12-hr dark photoperiod at 20°C in our central aquarium prior to any experimentation. Since the fish available locally (with GSI < 0.1%) were pre-pubertal and without sexual dimorphism based on external morphology, grass carp of mixed sexes were used in our pituitary cell preparation. During the process, the fish were anesthetized with MS222 (0.05%; Sigma-Aldrich, St. Louis, MO) followed by spinosectomy and routine procedures to obtain the pituitary from the sella turcica within the skull. The protocols for tissue sampling, pituitary cell preparation and associated *in vitro* experiments (CULATR 4608–18) were approved by the Committee for Animal Use in Teaching and Research at the University of Hong Kong (Hong Kong).

Grass carp GnRH2 (formerly called chicken GnRH-II/cGnRH-II) and GnRH3 (formerly called salmon GnRH/sGnRH) as well as the superactive GnRH analog in fish models, namely [D-Arg^6^, Pro^9^, NEt]-sGnRH (sGnRH-A), were synthesized by Genscript Biotech (Piscataway, NJ). Forskolin, MDL 12330A, H89, KG-501, C646, TPA, 1,2-dioctanoyl-*sn*-glycerol (DiC8), calphostin C, U73122, 2-APB, Bay K8644, KN62, nicardipine, calmidazolium, cyclosporin A, INCA-6 and 11R-VIVIT were obtained from Merck (Rahway, NJ) while 8-bromo-cAMP (8Br.cAMP), N^6^-benxoyl-cAMP (6-Bnz-cAMP), 8-pCPT-2’-O-Me-cAMP-AM (pCPT-O-Me-cAMP) and SGC-CBP30 were purchased from Tocris Bioscience (Bristol, UK). Except for the native GnRHs and sGnRH-A, which were dissolved in double-distilled deionized water, the other test substances were dissolved in DMSO and stored frozen at -80°C in small aliquots. On the day for drug testing in cell culture, frozen stocks of test substances were thawed on ice and diluted with prewarmed culture medium to target concentrations 15 min prior to drug administration. The final dilutions of DMSO were always ≤ 0.1% and did not alter PRL release and transcript expression based on our previous validation in carp pituitary cells (55).

### 2.2 Static incubation of grass carp pituitary cells

Grass carp pituitary cells prepared with trypsin/DNase II digestion method (56) were seeded in poly-D-lysine coated 24-well plates at a density of ~2.5 × 10^6^ cells/well and incubated for 18 hr at 28°C under 5% CO_2_ and saturated humidity in carp MEM (MEM with 26 mM NaHCO_3_, 25 mM HEPES and 1% antibiotic-antimycotic; pH 7.7) with 5% FBS to allow for recovery from enzyme digestion. After that, culture medium was replaced with serum-free carp MEM with 0.1% BSA containing (i) GnRHs/sGnRH-A for the duration/doses as indicated, (ii) pharmacological activators targeting different pathways, or (iii) sGnRH-A with co-treatment of pharmacological inhibitors for different signalling targets. For the doses of pharmacological tools used, they were selected based on our previous reports using the same activators/inhibitors to probe the respective pathways in pituitary cells of fish origin, e.g. in goldfish (26 & references within) and grass carp (57, 58), or using the effective dose range and ED_50_/IC_50_ reported for the respective drugs provided by the suppliers as a reference. After treatment, culture medium was harvested for monitoring PRL secretion using RIA with antiserum for carp PRL (59). For cell content and total production of PRL, pituitary cells were lysed in double-distilled deionized water with 3 cycles of freezing and thawing followed by sonication in a digital ultrasonic bath. After centrifugation at 10,000 ×*g* for 15 min at 4°C for sample clearing, the lysate obtained was used for RIA to determine PRL cell content and total production of PRL was defined as the sum total of PRL release and PRL cell content in the same sample. For PRL mRNA expression, total RNA was isolated from pituitary cells using TRIzol (Thermo Fisher, Waltham, MA), reversely transcribed by Superscript II (Thermo Fisher) and subjected to real-time PCR for PRL mRNA measurement. In selected experiments, parallel quantitation of CaM mRNA expression using a similar approach was also conducted. In both cases, real-time PCR was performed with a QuantiTect RT-PCR Kit (Qiagen, Hilden, Germany) in RotorGene-Q qPCR System (Qiagen) with primers and PCR conditions as described in **Supplementary Table S1**. The authenticity of PCR products was routinely confirmed after real-time PCR assays by melting curve analysis (with *Tm* at 93.6°C for PRL and 89.8°C for CaM) and serial dilutions of plasmid DNA carrying the ORF of target gene were used as the standards for data calibration. In these studies, real-time PCR for 18S RNA was also conducted to serve as the internal control.

### 2.3 GnRHR expression in grass carp lactotrophs

In grass carp, four types of GnRHR, namely GnRHR_1–4_, have been identified in the brain (35). To study GnRHR_1–4_ expression in the hypothalamo-pituitary axis, RT-PCR was conducted in total RNA prepared from the carp hypothalamus, pituitary and freshly dispersed pituitary cells. Similar to real-time PCR, RNA samples isolated with TRIzol were reversely transcribed and subjected to PCR with primers for GnRHR_1–4_ respectively using the PCR conditions described in **Supplementary Table S2**. To confirm GnRHR expression in lactotrophs, RT-PCR was also performed in immuno-identified lactotrophs isolated by Laser Capture Microdissection (LCM) (60). Briefly, immunocytochemical (ICC) staining with an antiserum for carp PRL using the ABC technique (56) was conducted in cytospin preparation of carp pituitary cells seeded at a density of ~5 × 10^4^ cells/slide and lactotrophs with distinct signal of PRL immunoreactivity were isolated by LCM with infra-red laser (7.5 μm in diameter) set at 65 mW and pulse duration at 1.2 ms using a PixCell Cell Isolation Workstation (Arcturus, Mountain View, CA). A total of 250 lactotrophs captured on Capsure HS caps (Arcturus) was dissolved in TRIzol for total RNA isolation. After DNase I digestion, half of the RNA sample was reversely transcribed with Superscript II (as “+RT” group) followed by PCR detection of GnRHR_1–4_ as described in preceding section but with the cycle no increased to 55 cycles. To check for possible contamination of genomic DNA, the remaining half of the RNA sample was subjected to the same procedures in the absence of Superscript II (as “-RT” group). In our study for GnRHR expression, parallel PCR for β actin was also conducted to serve as the internal control and the PCR products obtained for the respective targets were resolved in 1% agarose gel and visualized with ethidium bromide staining.

### 2.4 cAMP and Ca^2+^ signals in pituitary cells prepared from rostral pars distalis

Since the rostral pars distalis (RPD) was previously shown to be the region in carp pituitary with cells composed mostly of lactotrophs (56), the RPD of carp pituitaries was manually dissected under a stereo-microscope and used for pituitary cell preparation. The cells obtained (referred to as the “RPD cells”) were seeded at a density of ~3 × 10^6^ cells/2 ml/dish in poly-D-lysine coated 35 mm petri dishes (Corning, NY). After 18-hr recovery at 28°C in carp MEM with 5% FBS, culture medium in individual dishes was replaced with 0.9 ml HEPES-buffered HBSS solution (Hanks Balanced Salt Solution with 26 mM NaHCO_3_, 25 mM HEPES and 1% antibiotic-antimycotic; pH 7.7) with 0.1% BSA and 0.1 mM IBMX. After a brief incubation for 10 min, treatment was initiated by adding 0.1 ml of 10× stock of GnRH prepared in the same medium. RPD cells were then incubated with GnRH for another 15 min and the culture medium was harvested for measurement of cAMP release. Meanwhile, the cell content of cAMP was extracted from the remaining cells with 1 ml ice-cold absolute ethanol. The samples collected were then freeze-dried in a lyophilizer and stored at -20°C until their cAMP content measured with a BioTrak [^125^ I] cAMP RIA Kit (Amersham, UK). In this experiment, total production of cAMP was defined as the sum total of cAMP cell content and cAMP release in the same sample during the test period.

To monitor Ca^2+^ signals in carp lactotrophs, Ca^2+^ florescence imaging was conducted in RPD cells seeded at a density of 1 × 10^4^ cells/coverslip on poly-D-lysine precoated Cell-Locate Cover Glass (Thermo Fisher) after 18-hr recovery at 28°C in carp MEM with 5% FBS as described previously (55). Prior to Ca^2+^ imaging, PRD cells were preloaded with the Ca^2+^-sensitive dye Fura-2/AM (5 μM; Molecular Probes, Eugene, OR) for 45 min in Krebs-Ringer Buffer (120 mM NaCl, 4.7 mM KCl, 0.7 mM MgSO_4_, 1.2 mM CaCl_2_, 10 mM glucose and 15 mM HEPES; pH 7.4). After that, the cells were rinsed and incubated at room temperature with Krebs-Ringer Buffer for 15 min to allow for de-esterification of Fura-2/AM in RPD cells. Detection of intracellular Ca^2+^ ([Ca^2+^]i) signals was conducted at room temperature with epifluorescence microscope linked with a PTI DeltaScan Ca^2+^ Imaging System (Photon Technology, West Sussex, UK). Emission was monitored at 510 nm with excitation wavelength alternating between 340 nm and 380 nm at 1-sec interval. During the process, test substances were applied gently by hand pipetting into the imaging chamber with RPD cells attached to Cell-Locate coverslip. For *post facto* identification of lactotrophs with Ca^2+^ responses, bright field picture of RPD cells was captured followed by Ca^2+^ imaging before and after drug testing. After that, ICC staining of RPD cells with PRL antiserum was used to locate the lactotrophs and matched with the cells with Ca^2+^ responses grid by grid according to the numeric labels of the grid matrix photo-etched on the Cell-Locate coverslip.

### 2.5 Western blot for transcription factors and CaM expressed in RPD cells

Similar to the preceding study for cAMP signals, RPD cells were cultured in 35 mm dishes at a seeding density of ~6 × 10^6^ cells/2 ml/dish and incubated with carp MEM containing test substances for the duration as indicated in individual experiments. After that, the cells were rinsed with PBS and lysed in 250 μl RIPA lysis buffer (50 mM Tris-HCl, 1% Nonidet-P40, 0.25% sodium deoxycholate, 1 mM EDTA and 150 mM NaCl) supplemented with 1× protease and phosphatase inhibitor cocktail (Roche, Basel, Switzerland). The resulting lysate was cleared by centrifugation at 10,000 ×*g* at 4°C for 10 min and the supernatant obtained was resolved in 12% gel by SDS-PAGE followed by transblotting onto a nitrocellulose membrane (Thermo Fischer) using a TE77 Semi-dry Electroblotting Unit (Asmersham). After that, the membrane with protein samples was used for Western blot as described previously (60) using the antibodies for phosphorylated CREB (1:1300, Santa Cruz), total CREB (1:1000, Santa Cruz), c-Fos (1:2000, Santa Cruz), c-Jun (1:1000, Merck), CaM (1:1000, Sigma-Aldrich), and phosphorylated NFAT_1_ (also called NFATC2) and NFAT_2_ (also called NFATC1) (1:1000, Thermal Fischer), respectively. Chemiluminescence signals for target proteins were detected with Immobilon Western Chemiluminescent Substrate (Sigma) and visualized using a ChemiDoc Imaging System (Bio-Rad, Hercules, CA). In these studies, parallel blotting of β actin using an Actin Ab-I Kit (Oncogene, Cambridge, MA) was also conducted to serve as the loading control. In the experiment for NFAT phosphorylation, the antibodies for total protein of NFAT_1_ and NFAT_2_ from the same company were found to be not useful in carp model and parallel blotting for tubulin was used as the internal control.

### 2.6 Immunofluorescence imaging for nuclear translocation of CREB

RPD cells were seeded at a density of ~1.3 × 10^6^ cells/0.3 ml/chamber in poly-D-lysine coated μ-Slide 8-Well Chambered Coverslip (ibidi GmbH, Grafelfing, Germany) and incubated for 18 hr with carp MEM containing 5% FBS at 28°C under 5% CO_2_ for recovery after cell dispersion. After that, the culture medium was gently aspired and replaced with carp MEM with 0.1% BSA containing GnRH or forskolin (as positive control). Following a 30-min incubation with test substances, the cells were fixed in 4% paraformaldehyde, rinsed with PBS, subjected to endogenous peroxidase inactivation with 3% H_2_O_2_ and background reduction using blocking reagent (Roche), and incubated overnight at 4°C with the antibody for total CREB (1:1000, Santa Cruz). On the next day, the cells were washed 3 times with TNT buffer (0.1 M Tris-HCl, 0.15 NaCl and 0.05% Tween 20; pH 7.5), incubated for 30 min at room temperature with GAR-HRP (1: 200; Biotium Inc., Fremont, CA), and exposed to CF488A-TSA (1:400; Biotium Inc.) for tyramide signal amplification for 10 min followed by DAPI staining. For qualitative assessment of CREB responses, fluorescence images of CREB related to DAPI signals in the nuclei were captured using LSM 980 Confocal Microscopy System (Carl Zeiss, Jem, Germany). For quantitation of CREB nuclear translocation, similar fluorescence images were captured with BioTek Cytation-1 Cell Imaging Multimode Reader (Agilent, Santa Clara, CA). Using the images of CREB and DAPI signals in the same visual field, the total amount of CREB fluorescence in the nuclei of RPD cells and percentage of RPD cells with nuclear translocation of CREB were determined by the Gen 5 Image Analysis Software (Agilent) using a filter mask for fluorescence detection in the nucleus constructed based on the DAPI signals.

### 2.7 Data normalization and statistical analysis

For PRL secretion and protein expression in pituitary cells, the raw data from RIA expressed as ng/ml for PRL released into culture medium and ng/×10^6^ cells for PRL cell content and total production were used directly for statistical analysis. For transcript expression of PRL and CaM, the raw data from real-time PCR expressed as femtomole transcript detected per ×10^6^ cells were deduced from the standard curve constructed by serial dilutions of plasmid DNA with ORF of the target gene using data calibration under unsupervised mode with RotorGene-Q software 1.7 (Qiagen). The data obtained were normalized with 18S RNA detected in the same sample and transformed as a percentage of the mean value in the control group (“%Ctrl”). For dose-dependence of GnRH treatment on PRL secretion and PRL mRNA expression, the maximal responses and ED_50_ values of the respective dose-response curves were deduced by four-parameter logistic regression using Prism software (GraphPad, Boston, MA). For CREB fluorescence signal in the nucleus, the raw data in terms of relative fluorescence unit (as “RFU”) were normalized with DAPI signals detected in the same visual field. Data presented (Mean ± SEM, N = 3 - 4) were analysed with one-way ANOVA followed by Newman-Keuls test for dose-dependence studies and regular drug testing with a fixed duration of treatment or two-way ANOVA followed by Bonferroni test for time-course studies using Prism. Differences between experimental groups were considered as significant at *p* < 0.05.

## 3 Results

### 3.1 GnRHR expression in carp lactotrophs

**Figure 1:**
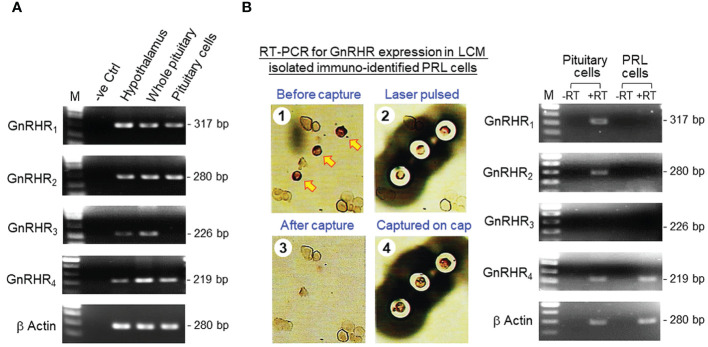
GnRHR expression in carp lactotrophs. **(A)** GnRHR expression in the hypothalamo-pituitary axis of grass carp. Using RT-PCR with primers for carp GnRHR_1–4_, the expression of the 4 isoforms of GnRHR in grass carp was examined in the hypothalamus, pituitary, and freshly dispersed pituitary cells. **(B)** GnRHR expression in immuno-identified lactotrophs (PRL cells) in grass carp. Pituitary cells with PRL signals as revealed by ICC staining using PRL antiserum were isolated using LCM and subjected to RT-PCR for GnRHR_1–4_ with (+RT) or without reverse transcription (-RT) during the preparation of RT samples. RT-PCR using mixed populations of pituitary cells was used as a positive control for our study (except for GnRHR_3_, which was used as a negative control). Parallel PCR for β actin expression was routinely conducted to serve as an internal control for the quality of RNA prepared.

Recently, grass carp was shown to have four GnRHRs, namely GnRHR_1–4_, expressed in a tissue-specific manner (35). To test for GnRHR_1–4_ expression in the hypothalamo-pituitary axis, RT-PCR was performed in the hypothalamus, pituitary and pituitary cells of grass carp. As shown in **Figure 1A**, transcript signals for the four types of GnRHR were detected in the hypothalamus and pituitary. Interestingly, the results based on pituitary cells were at variance with that of the whole pituitary. In this case, GnRHR_3_ expression was not apparent in pituitary cells despite the PCR signals for GnRHR_1, 2 & 4_ could still be noted. To confirm that lactotrophs can serve as a regulatory target for GnRH, RT-PCR for GnRHR_1–4_ was also examined in pituitary cells isolated by LCM with positive staining of PRL immunoreactivity (**Figure 1B**). Unlike the results for mixed populations of pituitary cells, only the PCR signal for GnRHR_4_ could be detected in immuno-identified lactotrophs captured by LCM.

### 3.2 GnRH-induced PRL release and gene expression in carp pituitary cells

**Figure 2:**
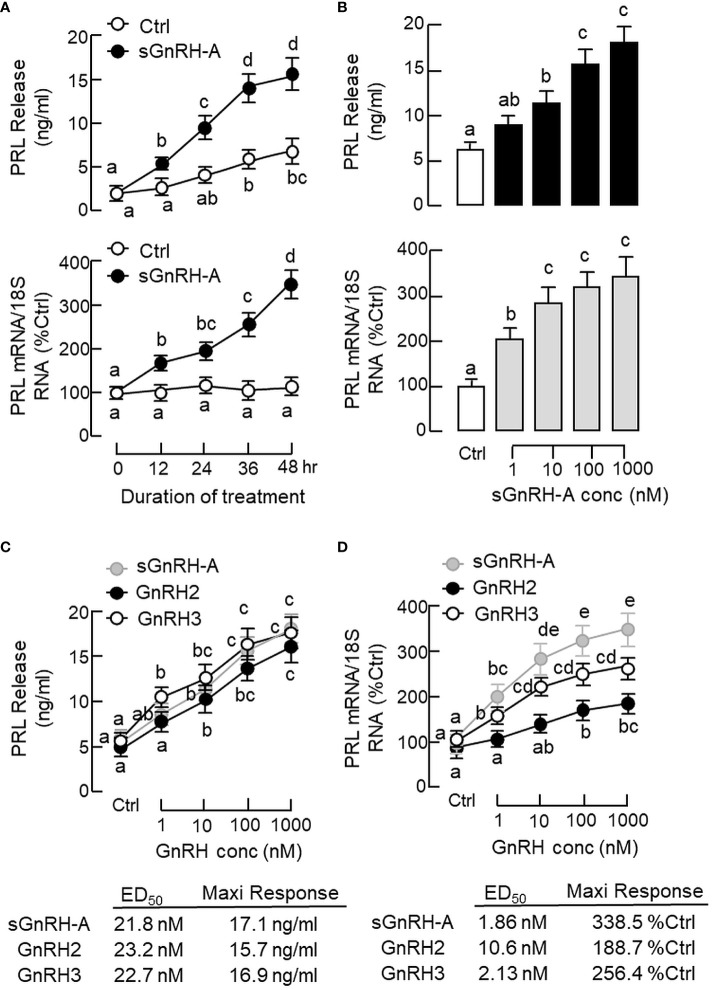
Effects of GnRH on PRL release and PRL mRNA expression in grass carp pituitary cells. **(A)** Time course and **(B)** dose dependence of sGnRH-A on PRL secretion and gene expression. Pituitary cells were exposed to sGnRH-A (1 μM) for the duration as indicated for the time course study or treated for 48 hr with increasing levels of sGnRH-A (1–1000 nM) for the dose-dependence study. Comparison of the potency and efficacy of sGnRH-A, GnRH2 and GnRH3 on **(C)** PRL release and **(D)** PRL mRNA expression at pituitary cell level. Pituitary cells were treated for 48 hr with increasing doses (1–1000 nM) of sGnRH-A, GnRH2 and GnRH3, respectively. The ED_50_ (for potency) and maximal responses (for efficacy) for individual dose-response curves were deduced by four-parameter logistic regression using GraphPad Prism. Experimental groups denoted by different letters represent a significant difference at *p* < 0.05 (one-way ANOVA followed by Newman-Keuls test for dose-dependence studies and two-way ANOVA followed by Bonferroni test for time-course study).

**Figure 3:**
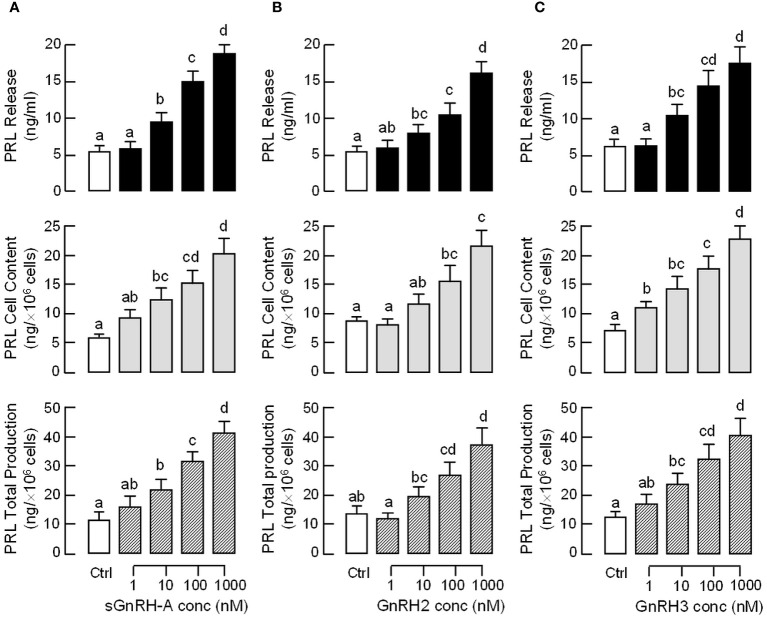
Effects of GnRH on PRL release, cell content and total production in grass carp pituitary cells. Pituitary cells were treated for 48 hr with increasing doses (1–1000 nM) of **(A)** sGnRH-A, **(B)** GnRH2 and **(C)** GnRH3, respectively. Total production of PRL was defined as the sum total of PRL cell content and PRL released into culture medium in the same sample. Experimental groups marked by different letters denote a significant difference at *p* < 0.05 (one-way ANOVA followed by Newman-Keuls test).

To test the pituitary action of GnRH on PRL regulation, static incubation of pituitary cells prepared from carp pituitaries was conducted with sGnRH-A treatment. The “superactive analog” sGnRH-A was used in our study as it can bind fish GnRHR with high affinity (61) and is commonly used in induced spawning for commercial fish (62). In carp pituitary cells, sGnRH-A (1 μM) could elevate PRL release and PRL mRNA levels in a time-related fashion up to 48 hr (**Figure 2A**). By fixing the duration of treatment at 48 hr, increasing concentrations of sGnRH-A (1–1000 nM) were found to induce PRL secretion and PRL gene expression in a dose-dependent manner (**Figure 2B**). The dose dependence of PRL responses caused by sGnRH-A could also be mimicked by parallel treatment with increasing levels (1–1000 nM) of GnRH2 and GnRH3, respectively (**Figures 2C, D**). Although the stimulatory effects of GnRH2 and GnRH3 on PRL secretion were comparable to sGnRH-A in terms of the maximal responses and ED_50_ values, the corresponding responses for PRL mRNA were quite different. In this case, the potency (reflected by ED_50_ values) and efficacy (revealed by maximal responses) for PRL gene expression induced by sGnRH-A and GnRH3 were notably higher than those of GnRH2. Consistent with the results on PRL mRNA expression, increasing levels (1–1000 nM) of sGnRH-A, GnRH2 and GnRH3 were also effective in elevating PRL cell content ant total production along with their stimulatory effects on PRL release (**Figures 3A–C**).

### 3.3 AC/cAMP/PKA pathway in GnRH-induced PRL release and gene expression

**Figure 4:**
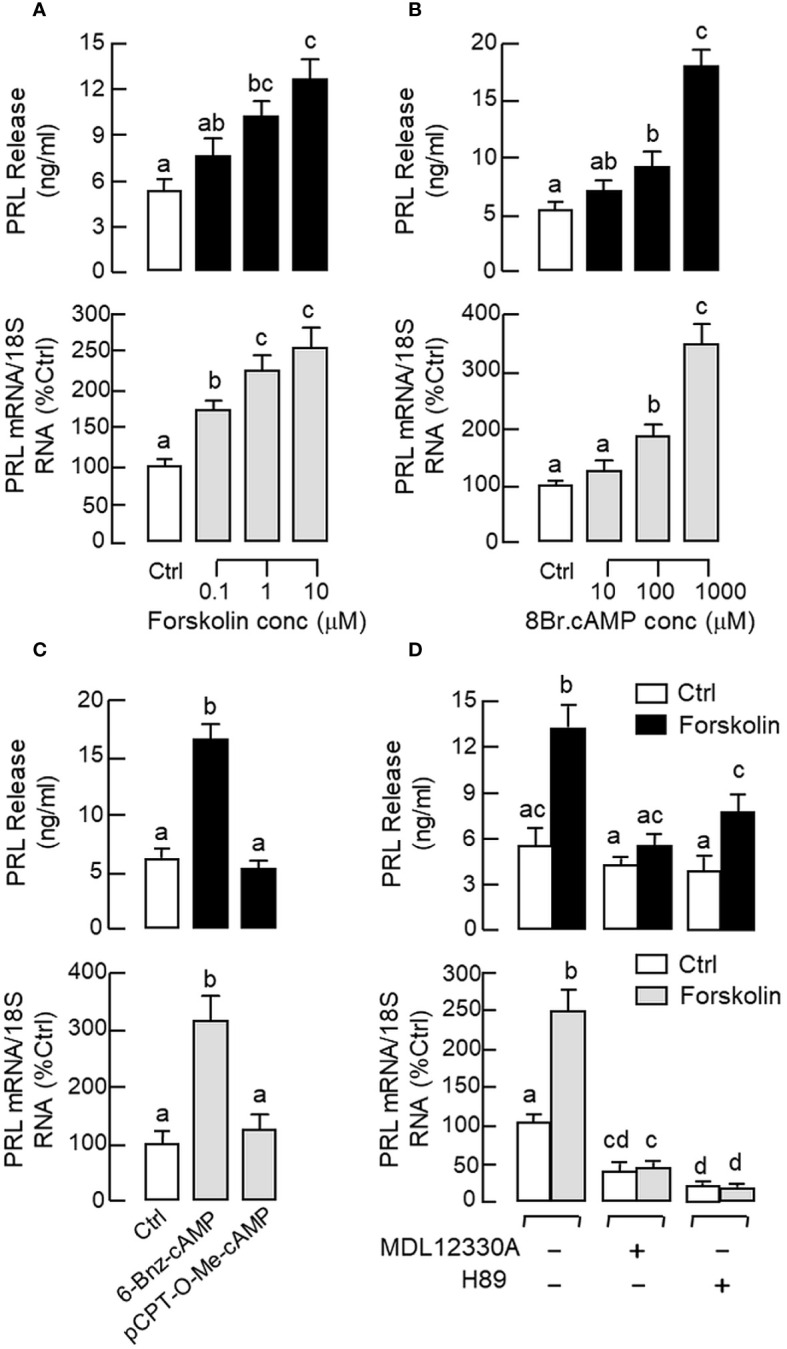
Functional role of cAMP/PKA-dependent pathway on PRL release and PRL mRNA expression in carp pituitary cells. Increasing cAMP at cellular level using **(A)** the AC activator forskolin (0.1–10 μM) or **(B)** cell-permeant cAMP analog 8Br.cAMP (10–1000 μM) on PRL release and gene expression. **(C)** Effects of the PKA-specific cAMP analog 6-Bnz-cAMP (1 mM) and Epac-specific cAMP analog pCPT-O-Me-cAMP (1 mM) on PRL secretion and gene expression. **(D)** Co-treatment with the AC inhibitor MDL 12330A (20 μM) or PKA blocker H89 (20 μM) on PRL secretion and gene expression induced by forskolin (10 μM). In these experiments, the duration of drug treatment was fixed at 48 hr. Experimental groups denoted by difference letters represent a significant difference at *p* < 0.05 (one-way ANOVA followed by Newman-Keuls test).

Given that cAMP involvement in GnRH-induced LH secretion and gene expression has been reported in fish models (63), the functional role of cAMP/PKA pathway in PRL regulation was examined in carp pituitary cells. Similar to the results of GnRH treatment, a 48-hr incubation with the adenylate cyclase (AC) activator forskolin (0.1–10 μM, **Figure 4A**) and membrane-permeant cAMP analog 8Br.cAMP (10–1000 μM, **Figure 4B**) were both effective in increasing PRL release and PRL mRNA levels in a dose-dependent manner. Since PKA and Epac are known to be the downstream effectors for cAMP, their possible involvement in PRL regulation was also tested. As shown in **Figure 4C**, PRL secretion and gene expression could be elevated by the PKA-specific cAMP analog 6-Bnz-cAMP (1 mM) but not the Epac-specific cAMP analog pCPT-O-Me-cAMP (1 mM). In carp pituitary cells, the stimulatory effects of forskolin (10 μM) on PRL secretion and transcript expression could also be reduced/totally blocked by co-treatment with the AC inhibitor MDL 12330A (20 μM) or PKA inactivator H89 (20 μM) (**Figure 4D**).

**Figure 5:**
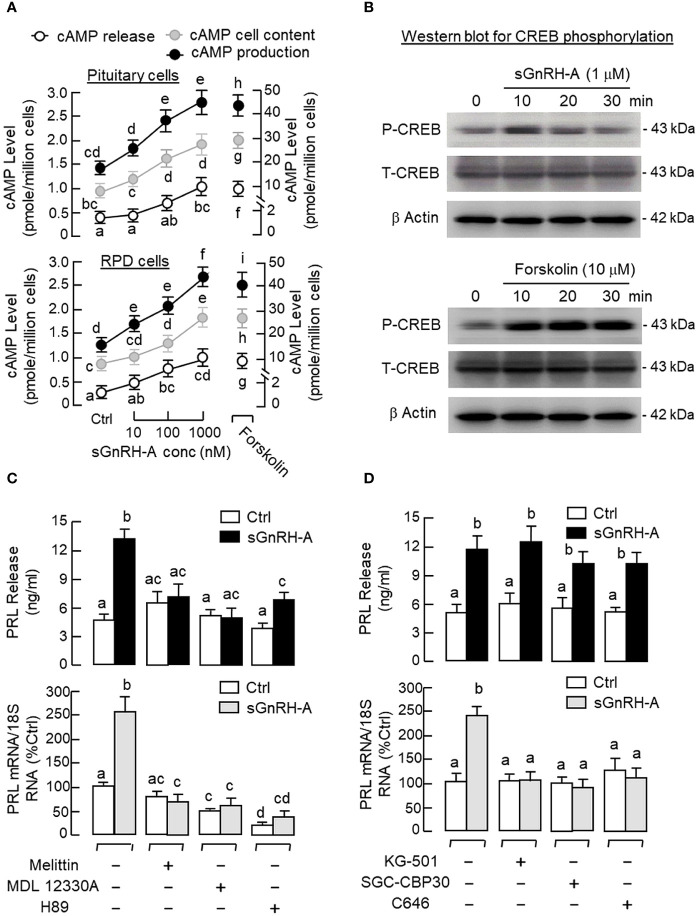
Functional role of cAMP/PKA-dependent pathway on GnRH-induced PRL secretion and PRL mRNA expression in carp pituitary cells. **(A)** GnRH treatment on cAMP production in carp RPD cells. RPD cells prepared from the RPD of carp pituitaries were treated for 15 min with sGnRH-A (10–1000 nM) with parallel monitoring of cAMP release and cAMP cell content. cAMP production was defined as the sum of cAMP cell content and cAMP released into culture medium in the same sample. Similar treatment with sGnRH-A was also performed in pituitary cells prepared from whole pituitaries as a parallel control. In the same study, forskolin treatment (10 μM) was used as a positive control for cAMP production at cellular level. **(B)** GnRH treatment on CREB phosphorylation in carp RPD cells. RPD cells were exposed to sGnRH-A (1 μM) up to 30 min followed by Western blot using the antibodies for phosphorylated form (“P”-form) and total protein (“T”-form) of CREB. Forskolin treatment (10 μM) was used as a positive control and parallel Western blot for β actin was used as the loading control for individual experiments. Blocking **(C)** cAMP/PKA and **(D)** CREB/CBP signalling on GnRH-induced PRL secretion and gene expression in carp pituitary cells. Pituitary cells were incubated with sGnRH-A (1 μM) for 48 hr with/without the co-treatment the G_s_ inhibitor melittin (100 nM), AC repressor MDL 12330A (20 μM), PKA blocker H89 (20 μM), CREB inactivator KG-501 (10 μM), CREB: CBP interaction inhibitor SGC-CBP30 (10 μM), and HAT blocker C646 (0.1 μM), respectively. Groups denoted by different letters represent a significant difference at *p* < 0.05.

**Figure 6:**
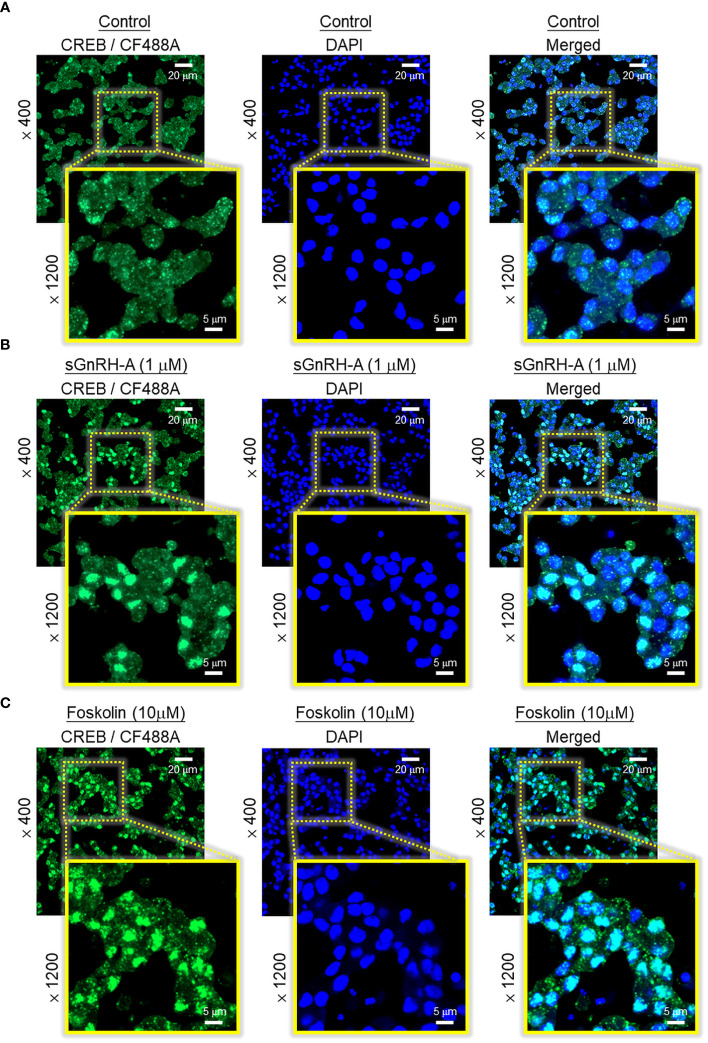
Effect of GnRH treatment on CREB nuclear translocation in carp RPD cells. RPD cells cultured in μ-Slide 8-chambered coverslips were treated for 30 min with **(A)** culture medium (as control group), **(B)** sGnRH-A (1 μM), or **(C)** forskolin (10 μM, as a positive control) followed by immunofluorescence staining with the antibody for total CREB. Using confocal imaging, CREB signals revealed by CF488A fluorescence and DAPI signals (to define the nuclei of RPD cells) were captured and these images were merged with the Zen lite 2.1 software to highlight the nuclei with notable signals of nuclear translocation of CREB after drug treatment.

In pituitary cells prepared from whole pituitaries (**Figure 5A**, upper panel) or RPD cells prepared from the RPD region of carp pituitaries (**Figure 5A**, lower panel), short-term incubation for 15 min with increasing doses of sGnRH-A (10–1000 nM) was found to up-regulate cAMP release, cAMP cell content and total production of cAMP in a dose-dependent manner. Notable responses for cAMP secretion and cAMP production were also observed with similar treatment of forskolin (10 μM), which was used as a positive control in our study. In agreement with protein phosphorylation by PKA via cAMP signals, similar treatment with sGnRH-A (1 μM) could induce a rapid but transient rise in CREB phosphorylation in RPD cells with a peak response at 10 min (**Figure 5B**, upper panel). In the same experiment, CREB phosphorylation also occurred with forskolin treatment (10 μM) and the response was sustained up to 30 min (**Figure 5B**, lower panel). Using fluorescence imaging after ICC staining, a 30-min incubation with sGnRH-A (1μM) and forskolin (10 μM) were both effective in triggering a notable rise in nuclear translocation of CREB in RPD cells (**Figure 6**). The results obtained were also consistent with parallel fluorescence quantitation and cell counting using a Cytation-1 imaging station, in which CREB signals detected in the nucleus and percentage of RPD cells with nuclear translocation of CREB were found to be significantly elevated by sGnRH-A and forskolin in the same study (**Supplementary Figure S2**). In parallel studies with pituitary cells prepared from whole pituitaries, PRL release and PRL mRNA expression induced by sGnRH-A (1 μM) could be totally negated by blocking G_s_ activation with the G_s_ inhibitor melittin (100 nM). Similar to preceding study with forskolin, the PRL responses caused by sGnRH-A (1 μM) could be reduced/abolished by co-treatment with the AC inhibitor MDL 12330A (20 μM) or PKA inhibitor H89 (20 μM) (**Figure 5C**). Of note, PRL gene expression induced by sGnRH-A (1 μM) were also blocked by similar treatment with the CREB inhibitor KG-501 (10 μM), CREB:CBP interaction blocker SGC-CBP30 (10 μM) or histone acetyltransferase (HAT) inactivator C646 (0.1 μM). Interestingly, these inhibitors were not effective in altering the parallel responses for PRL secretion (**Figure 5D**).

### 3.4 PLC/IP_3_/PKC pathway in GnRH-induced PRL release and gene expression

**Figure 7:**
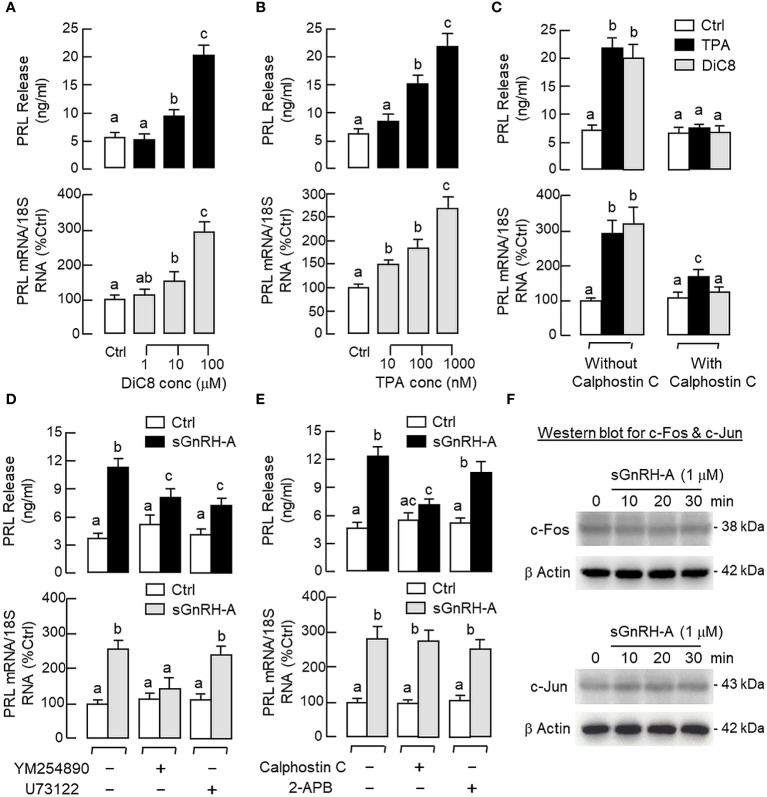
Functional role of PLC/PKC-dependent pathway on PRL release and PRL mRNA expression in carp pituitary cells. To test the effect of PKC activation on PRL release and gene expression, pituitary cells were treated for 48 hr with **(A)** the DAG analog DiC8 (1–100 μM) or **(B)** PKC activator TPA (10–1000 nM). **(C)** PKC involvement in the PRL responses caused by TPA (1 μM) and DiC8 (100 μM) were further confirmed with co-treatment of the PKC inhibitor calphostin C (1 μM). In parallel experiments, the functional role of **(D)** G_q/11_ coupling with PLC and **(E)** IP_3_ and PKC working downstream of PLC in GnRH-induced PRL release and PRL mRNA expression was also examined. In this case, pituitary cells were exposed to sGnRH-A (1 μM) for 48 hr in the presence of the G_q/11_ blocker YM254890 (1 μM), PLC repressor U73122 (5 μM), PKC inhibitor calphostin C (1 μM) and IP_3_ channel inactivator 2-APB (50 μM), respectively. **(F)** Possible link of AP-1 transcription factors with GnRH treatment in carp RPD cells. RPD cells were challenged with sGnRH-A (1 μM) up to 30 min followed by Western blot using the antibodies for c-Fos and c-Jun, respectively. Western blot for β actin was also conducted to serve as a loading control. For PRL secretion and gene expression in carp pituitary cells, experimental groups denoted by different letters represent a significant difference at *p* < 0.05.

Given that the PLC/IP_3_/PKC pathway is well-documented to be a major component of GnRH signalling for LH secretion and gene expression in mammalian models (24, 25), its involvement in PRL regulation by GnRH was also tested in carp pituitary cells. As shown in **Figures 7A, B**, a 48-hr incubation with increasing levels of the membrane-permeant DAG analog DiC8 (1–100 μM) or PKC activator TPA (10–1000 nM) was effective in elevating PRL release and PRL mRNA levels in a dose-related fashion. Furthermore, the PRL responses induced by TPA (1 μM) and DiC8 (100 μM) could be reduced/negated by co-treatment with the PKC inhibitor calphostin C (1 μM, **Figure 7C**). In parallel studies with sGnRH-A as the stimulant (**Figures 7D, E**), co-treatment with the G_q/11_ blocker YM254890 (1 μM), PLC blocker U73122 (5 μM) or PKC inhibitor calphostin C (1 μM) was found to reduce PRL release induced by sGnRH-A (1 μM). Inhibiting G_q/11_ with YM254890 (1 μM) was also effective in blocking the PRL transcript response caused by sGnRH-A (1 μM) but no major effect could be noted for the corresponding stimulation on PRL gene expression with U73122 (5 μM) or calphostin C treatment (1 μM). In the same study, co-treatment with the IP_3_ channel inhibitor 2-APB (50 μM) did not alter sGnRH-A stimulation on PRL secretion and gene expression (**Figure 7E**), and the same was true with co-treatment of the IP_3_ antagonist xestospongin C (3 mM) (data not shown). To examine the possible role of transcription factors working downstream of the PLC/PKC pathway in PRL regulation by GnRH, Western blot for c-Fos and c-Jun was conducted in RPD cells with GnRH stimulation. As shown in **Figure 7F**, notable changes for c-Fos and c-Jun expression in RPD cells were not apparent with sGnRH-A treatment (1 μM) up to 30 min.

### 3.5 Ca^2+^/CaM-dependent pathway in GnRH-induced PRL release and gene expression

**Figure 8:**
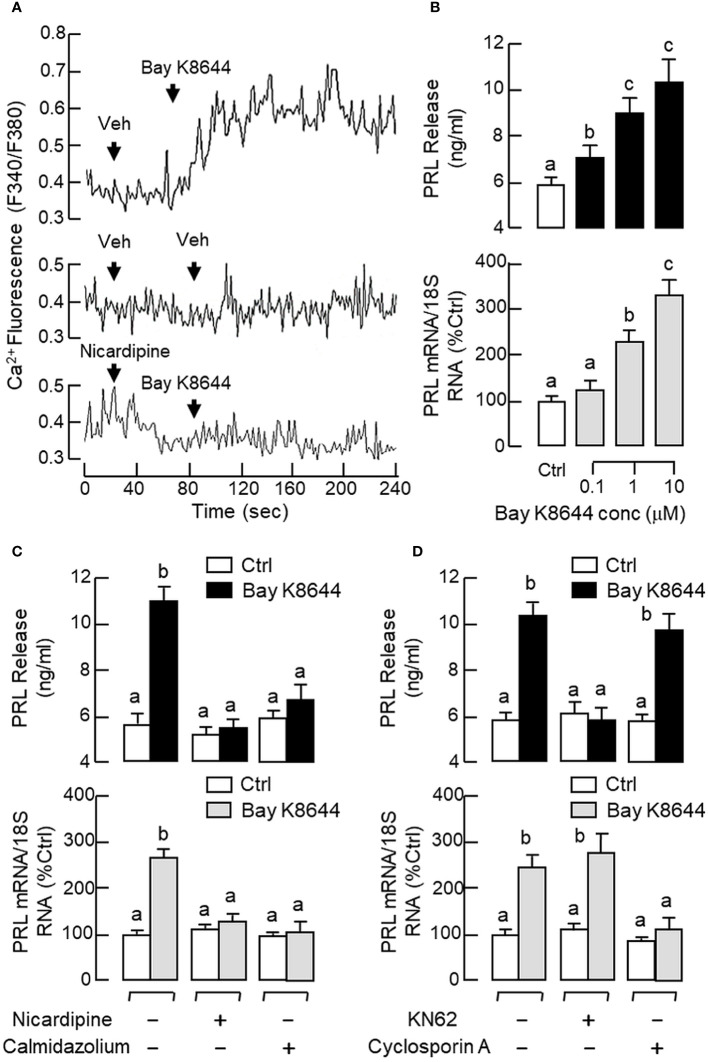
Functional role of Ca^2+^/CaM-dependent pathway on PRL release and PRL mRNA expression in carp pituitary cells. **(A)** Activation of L-type VSCC on Ca^2+^ signals detected in carp RPD cells. RPD cells preloaded with the Ca^2+^-sensitive dye Fura-2 were exposed to the VSCC activator Bay K8644 (10 μM) in the presence or absence of the L-type VSCC blocker nicardipine (5 μM). During the process, the kinetics of Ca^2+^ response was monitored based on the ratio of fluorescence output with excitation wavelength alternating between 340 nm and 380 nm (as “F340/F380”). In this experiment, the vehicle (Veh) used for solubilization of test substances was used as the control treatment. **(B)** VSCC activation in carp pituitary cells by increasing doses of Bay K8644 (0.1–10 μM) on PRL release and PRL mRNA expression. **(C)** L-type VSCC blockade and CaM antagonism, or **(D)** CaMK-II and CaN inactivation on the PRL responses induced by Bay K8644. Carp pituitary cells were challenged with Bay K8644 (5 μM) for 48 hr with/without the co-treatment of the L-type VSCC blocker nicardipine (5 μM), CaM antagonist calmidazolium (1 μM), CaMK-II inhibitor KN62 (5 μM) or CaN inactivator cyclosporin A (100 nM). For PRL secretion and gene expression in carp pituitary cells, experimental groups denoted by different letters represent a significant difference at *p* < 0.05.

To shed light on the role of Ca^2+^/CaM-dependent pathway in PRL regulation by GnRH, RPD cells were preloaded with the Ca^2+^-sensitive dye Fura-2 and used for single-cell Ca^2+^ imaging with GnRH treatment. As a first step for system validation, RPD cells with dye loading were challenged with the VSCC activator BayK 8644. As shown in **Figure 8A**, a rapid rise in [Ca^2+^]i signals were noted after BayK 8644 treatment (10 μM) and this Ca^2+^ response could be negated by the L-type VSCC blocker nicardipine (5 μM). In parallel studies with pituitary cells prepared from whole pituitaries, BayK 8644 (0.1–10 μM) was also effective in elevating PRL release and PRL mRNA levels in a dose-dependent manner (**Figure 8B**) and these stimulatory effects were ablated by co-treatment with the L-type VSCC blocker nicardipine (5 μM) or calmodulin (CaM) antagonist calmidazolium (1 μM) (**Figure 8C**). Of note, co-treatment with the Ca^2+^/CaM-dependent protein kinase-II (CaMK-II) inhibitor KN62 (5 μM) could abolish the PRL-releasing effect of BayK 8644 (5 μM) but not the corresponding induction on PRL gene expression whereas the opposite was true for parallel co-treatment with the calcineurin (CaN) inactivator cyclosporin A (100 nM) (**Figure 8D**).

**Figure 9:**
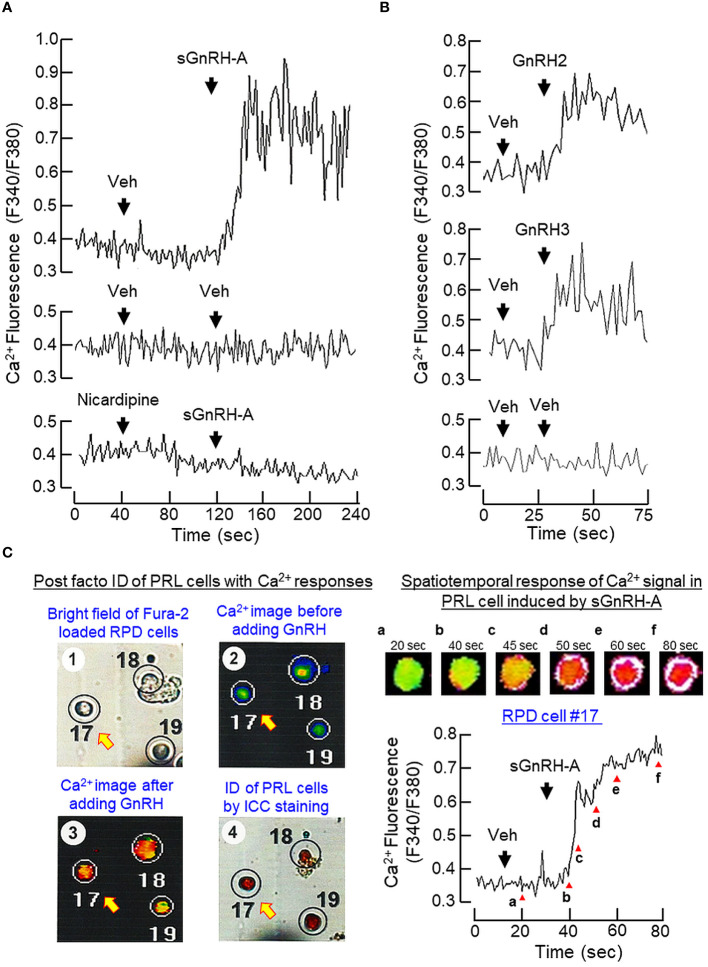
Ca^2+^ responses induced by GnRH in carp lactotrophs. **(A)** Inactivating L-type VSCC on Ca^2+^ signals induced by GnRH in carp RPD cells. RPD cells preloaded with Ca^2+^-sensitive dye Fura-2 were challenged with sGnRH-A (1 μM) in the presence or absence of the L-type VSCC blocker nicardipine (5 μM). **(B)** Effects of native GnRHs on Ca^2+^ signals in carp RPD cells. Using a similar approach, RPD cells were also treated with the native GnRHs found in grass carp, namely GnRH2 (1 μM) and GnRH3 (1 μM), respectively. During the process, the kinetics of Ca^2+^ response was monitored based on the ratio of fluorescence output with excitation wavelength alternating between 340 nm and 380 nm (as “F340/F380”). **(C)** Ca^2+^ responses induced by GnRH in immuno-identified lactotrophs. RPD cells cultured on Cell-Locate coverslips were preloaded with Fura-2 and challenged with sGnRH-A (1 μM) with parallel fluorescence imaging of Ca^2+^ responses. After that, *post facto* identification of lactotrophs (PRL cells) among the RPD cells attached on the same coverslip was conducted by ICC staining using the antiserum for carp PRL and mapped with the responsive cells with Ca^2+^ rise induced by sGnRH-A. The *post facto* identification of lactotrophs with Ca^2+^ responses was shown in the left panels while the kinetics of Ca^2+^ responses observed in a representative PRL cell (PRD cell #17), with Ca^2+^ images captured at time points as indicated by the letters in lower case) was shown in the right panels.

**Figure 10:**
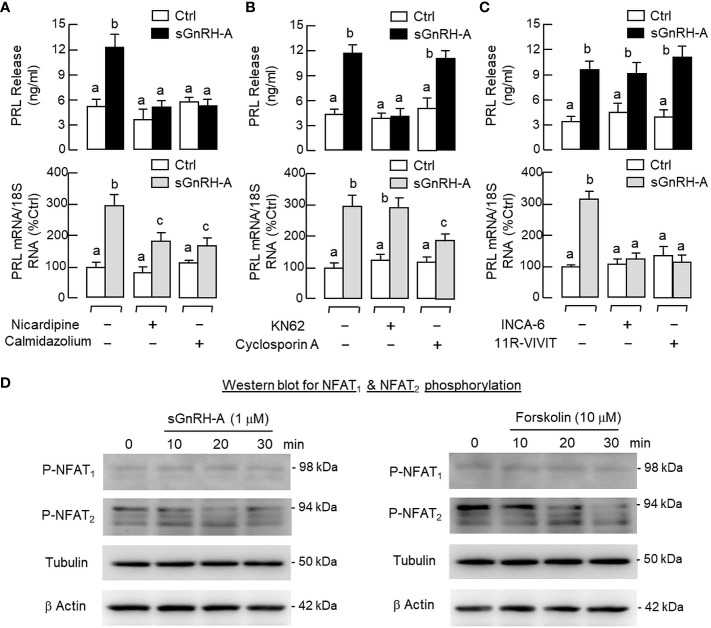
Functional role of Ca^2+^/CaM-dependent pathway on GnRH-induced PRL release and PRL mRNA expression in carp pituitary cells. **(A)** L-type VSCC blockade and CaM antagonism, **(B)** CaMK-II inhibition and CaN inactivation, and **(C)** interfering CaN: NFAT interaction or nuclear translocation of NFAT on PRL secretion and gene expression induced by GnRH. In these experiments, pituitary cells were treated with sGnRH-A (1 μM) for 48 hr in the presence or absence of the L-type VSCC inactivator nicardipine (5 μM), CaM antagonist calmidazolium (1 μM), CaMK-II inhibitor KN62 (5 μM), CaN repressor cyclosporin A (100 nM), CaN: NFAT interaction blocker INCA-6 (5 μM) and NFAT nuclear translocation inhibitor 11R-VIVIT (1 μM), respectively. Groups denoted by different letters represent a significant difference at p < 0.05. **(D)** Effects of GnRH and forskolin treatment on NFAT dephosphorylation in carp RPD cells. RPD cells were challenged with sGnRH-A (1 μM) or forskolin (10 μM) up to 30 min followed by Western blot using the antibodies for phosphorylated form (“P”-form) of NFAT_1_ and NFAT_2_, respectively. Given that no specific signals could be detected using the antibodies for total protein of NFAT_1_ and NFAT_2_ provided by the company, Western blot for tubulin was used as the internal control along with the parallel blotting for β actin as loading control.

Similar to the Ca^2+^ responses caused by BayK 8644, a rapid rise in [Ca^2+^]i could be induced by sGnRH-A (1 μM) in RPD cells and this Ca^2+^ response was sensitive to the blockade by the L-type VSCC inhibitor nicardipine (5 μM) (**Figure 9A**). In RPD cells, the stimulatory effect of sGnRH-A on [Ca^2+^]i could be mimicked by parallel treatment with GnRH2 (1 μM) and GnRH3 (1 μM), respectively (**Figure 9B**). To confirm that the Ca^2+^ responses indeed can occur in carp lactotrophs, *post facto* identification of lactotrophs by ICC staining with PRL antiserum was performed in RPD cells after Ca^2+^ imaging with sGnRH-A (1 μM, **Figure 9C**). In this case, RPD cells with PRL immunoreactivity were shown to have notable Ca^2+^ responses induced by sGnRH-A. The Ca^2+^ signals were initiated in peripheral region underneath the plasma membrane, with the highest Ca^2+^ rise occurred in the subplasmalemmal space and spread into the inner core of the responsive cells. As shown in **Figure 10A**, sGnRH-A (1 μM) consistently up-regulated PRL release and PRL mRNA levels in carp pituitary cells and these PRL responses could be reduced/abolished by the L-type VSCC blocker nicardipine (5 μM) or calmodulin (CaM) antagonist calmidazolium (1 μM). Similar to BayK 8644, PRL release induced by sGnRH-A (1 μM) could be blocked by the CaMK-II inhibitor KN62 (5 μM) but not the CaN inactivator cyclosporin A (100 nM). However, the corresponding rise in PRL mRNA level was reduced by cyclosporin A but not KN62 (**Figure 10B**). In parallel study to examine the possible involvement of the transcription factor NFAT working downstream of CaN, PRL transcript expression induced by sGnRH-A (1 μM) but not the corresponding rise in PRL secretion was found to be totally negated by co-treatment with the CaN:NFAT interaction inhibitor INCA-6 (5 μM) or NFAT nuclear translocation blocker 11R-VIVIT (1 μM) (**Figure 10C**). As revealed by Western blot, phosphorylated forms of NFAT_1_ and NFAT_2_ could be detected in RPD cells, probably with NFAT_2_ as the major form. In the same study, treatment of RPD cells with sGnRH-A (1 μM) up to 30 min did not alter the phosphorylation status of NFAT_1_ but induced a transient drop in NFAT_2_ phosphorylation with a peak at 20 min (**Figure 10D**, left panel). Forskolin treatment (10 μM) also reduced NFAT_2_ phosphorylation in RPD cells but with no effect on the corresponding signals of NFAT_1_, and this inhibitory action became quite noticeable in a time-dependent manner up to 30 min (**Figure 10D**, right panel).

### 3.6 Coupling of Ca^2+^/CaM-dependent pathway with cAMP/PKA signalling in PRL regulation

**Figure 11:**
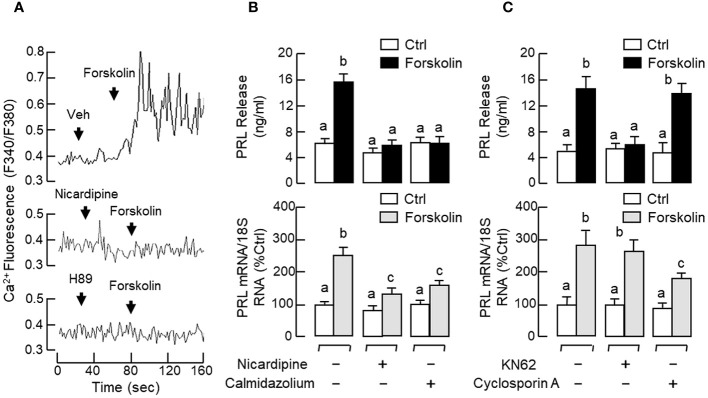
Functional coupling of cAMP/PKA-dependent pathway with Ca^2+^/CaM-dependent signalling on PRL release and PRL mRNA expression in carp pituitary cells. **(A)** Ca^2+^ entry through L-type VSCC induced by forskolin via PKA activation in carp RPD cells. RPD cells preloaded with the Ca^2+^ sensitive dye Fura-2 were challenged with the AC activator forskolin (10 μM) with/without the co-treatment of the L-type VSCC blocker nicardipine (5 μM) or PKA inactivator H89 (20 μM). During the process, the kinetics of Ca^2+^ response was monitored based on the ratio of fluorescence output with excitation wavelength alternating between 340 nm and 380 nm (as “F340/F380”). **(B)** L-type VSCC blockade and CaM antagonism, and **(C)** CaMK-II and CaN inactivation on PRL release and PRL mRNA expression induced by forskolin in carp pituitary cells. In these experiments, pituitary cells were treated for 48 hr with forskolin (10 μM) in the presence or absence of the L-type VSCC inactivator nicardipine (5 μM), CaM antagonist calmidazolium (1 μM), CaMK-II inhibitor KN62 (5 μM), or CaN repressor cyclosporin A (100 nM). Groups denoted by different letters represent a significant difference at p < 0.05.

**Figure 12:**
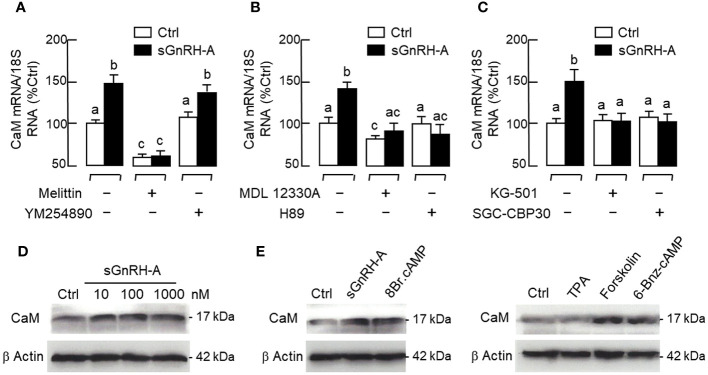
CaM expression induced by GnRH via cAMP/PKA-dependent pathway in carp RPD cells. **(A)** Signal coupling via G_s_ and G_q/11_, **(B)** Blockade of AC and PKA, and **(C)** Inhibiting CREB or interfering CREB: CBP interaction on CaM mRNA expression induced by GnRH. RPD cells were treated for 48 hr with sGnRH-A (1 μM) with/without the co-treatment of the G_s_ inactivator melittin (100 nM), G_q/11_ inhibitor YM254890 (1 μM), AC repressor MDA 12330A (20 μM), PKA inhibitor H89 (20 μM), CREB inactivator KG-501 (10 μM), or CREB: CBP interaction blocker SGC-CBP30 (10 μM). Groups denoted by different letters represent a significant difference at p < 0.05. **(D)** Increasing doses of GnRH stimulation, and **(E)** PKA and PKC activation on CaM protein expression in carp RPD cells. RPD cells were challenged for 48 hrs with increasing concentrations of sGnRH-A (10–1000 nM) or a fixed dose of sGnRH-A (1 μM), cell-permeable cAMP analog 8Br.cAMP (1 mM), PKC activator TPA (1 μM), AC activator forskolin (10 μM), and PKA-specific cAMP analog 6-Bnz-cAMP (1 mM), respectively. After treatment, cell lysate was prepared and subjected to Western blot using the antibody for CaM. Parallel Western blot for β actin was also conducted to serve as the loading control.

In our previous studies with carp pituitary cells, the GH-releasing effect mediated by cAMP-dependent pathway (e.g., induced by forskolin) was found to be Ca^2+^-dependent and required the entry of extracellular Ca^2+^ ([Ca^2+^]e) via VSCC (57). Since GH and PRL are evolved from the same ancestral gene, it would be of interest to know if a similar crosstalk between cAMP- and Ca^2+^-dependent cascades also plays a role in PRL regulation. As shown in **Figure 11A**, treatment with the AC activator forskolin (10 μM) could induce a rapid rise in [Ca^2+^]i in RPD cells and this Ca^2+^ response was totally abolished in the presence of the PKA inhibitor H89 (20 μM) or L-type VSCC blocker nicardipine (5 μM). In pituitary cells prepared from carp pituitaries, forskolin treatment (10 μM) consistently increased PRL secretion and PRL mRNA levels and these PRL responses could be reduced/obliterated by the L-type VSCC blocker nicardipine (5 μM) or CaM antagonist calmidazolium (1 μM) (**Figure 11B**). Similar treatment with the CaMK-II inhibitor KN62 (5 μM) could also block the PRL-releasing effect of forskolin (10 μM) but not the corresponding response on PRL transcript. However, parallel treatment with the CaN inactivator cyclosporin A (100 nM) was found to reduce the PRL gene expression caused by forskolin with no effect on its stimulation for PRL release (**Figure 11C**). Given that CaM expression at pituitary level was previously shown to be a modulatory target involved in GH (58) and PRL regulation in grass carp (55), the effect of GnRH on CaM expression was also examined in carp RPD cells. In this case, sGnRH-A treatment (1 μM) could up-regulate CaM mRNA levels in our cell model and this CaM response could be blocked by the G_s_ inhibitor melittin (100 nM) but not G_q/11_ inhibitor YM254890 (1 μM) (**Figure 12A**). Similar to the PRL responses in preceding study, the stimulatory effect of sGnRH-A (1 μM) on CaM gene expression was totally negated by co-treatment with the AC inhibitor MDL 12330A (20 μM), PKA repressor H89 (20 μM), CREB inactivator KG-501 (10 μM) or CREB:CBP interaction blocker SGC-CBP30 (10 μM) (**Figures 12B, C**). As shown by Western blot in RPD cells, increasing levels of sGnRH-A (10–1000 nM) was also effective in elevating protein expression of CAM in a dose-dependent manner (**Figure 12D**) and this stimulatory effect could be mimicked by parallel exposure to the AC stimulator forskolin (10 μM), cell-permeant cAMP analog 8Br.cAMP (1 mM), and PKA-specific cAMP analog 6-Bnz-cAMP (1 mM) but not the PKC activator TPA (1 μM) (**Figure 12E**).

## 4 Discussion

In vertebrate species, the pivotal role of GnRH in reproduction, especially via LH and FSH regulation, is well-conserved from fish to mammals (for review, see (5, 6)). In mammals, GnRH-induced PRL release has been reported *in vivo* in ovariectomized monkey (42) and in human female with HMG treatment during assisted reproduction (41). The *in vitro* studies for PRL regulation via pituitary action of GnRH, however, are not consistent and vary with sex, reproductive stages and neonatal development. For examples, PRL secretion induced by GnRH was demonstrated in pituitary fragments/pituitary cells prepared from human (64), sheep (43) and neonatal rat (65) but not in other species (e.g., in porcine pituitary cells) (44, 45). Of note, the PRL-releasing effect of GnRH was observed in pituitary cells prepared from female sheep during the breeding season but not in other time of the year (43). In male rats, PRL secretion was not affected by GnRH treatment (66) but GnRH-induced PRL release could be noted in pituitary cells prepared from female rats during the neonatal period of day 3–9 after birth (65). For the post-receptor signalling mediating PRL regulation by GnRH, the information available was based on GGH3 cell lines with functional expression of rat GnRHR. In this case, PRL release could be triggered by GnRH with a parallel rise in IP_3_ levels (46) and mediated by cAMP signals and protein synthesis coupled with G_s_ activation (47). Despite the fact that PRL also plays a key role in reproduction (see introduction), no consensus has been reached for the physiological role of PRL regulation by GnRH. For comparative studies on the same topic, only five reports have been published and they are all based on fish models, with two in tilapia, one in sockeye salmon, and the other two in masu salmon. For the studies in tilapia, *in vitro* culture of RPD/RPD cells prepared from male tilapia were used and the three native forms of GnRHs (with potency of GnRH2 > GnRH1 > GnRH3) were shown to induce PRL secretion (52) via PLC/IP_3_- and Ca^2+^-dependent mechanisms (51). For the reports in salmon, the focus was on pituitary gene expression and PRL mRNA levels in the pituitary of masu salmon (67) but not sockeye salmon (54) could be elevated by long-term implantation with GnRH. Similar to the study in sheep model, GnRH could up-regulate PRL mRNA expression in pituitary cells prepared from female masu salmon during pre-spawning phase but not in other reproductive stages (53). To date, the studies on signal transduction for the PRL-releasing effect of GnRH are rather limited and no information is available for the post-receptor signalling mediating the pituitary action of GnRH on PRL gene expression.

In fish models, nerve fibres with GnRH immunoreactivity are located in close proximity to lactotrophs (e.g., in perch) (68) and GnRHR expression can be detected in lactotrophs in the anterior pituitary (e.g., in tilapia) (38). These findings provide the initial support for lactotrophs as the target cells for GnRH within the fish pituitary. In grass carp, two forms of GnRH, GnRH2 and GnRH3, and four types of GnRHR of the GnRHR-II family, namely GnRHR_1–4_, have been cloned recently (35). In our study, GnRHR_1–4_ expression could be located in the hypothalamo-pituitary axis of grass carp, which is consistent with the role of GnRH as a hypophysiotropic factor for gonadotropin regulation in carp species. Of note, the transcript expression of GnRHR_1–4_ could be detected by RT-PCR in intact pituitary but the corresponding signal of GnRHR_3_ was missing in parallel study with freshly dispersed pituitary cells. Since the washing steps with cell pelleting by low-speed centrifugation used in our pituitary cell preparation (56) are known to remove cell debris and nerve fibres/terminals, we do not exclude the possibility that GnRHR_3_ is expressed in nerve fibres/terminals but not in endocrine cells within the carp pituitary. Using RT-PCR coupled to LCM capture of pituitary cells with PRL immunoreactivity, only the transcript signal for GnRHR_4_ but not the other GnRHRs was found in carp lactotrophs. In carp pituitary cells prepared from whole pituitaries, PRL release, PRL mRNA level and PRL protein production could be elevated by sGnRH-A, a GnRH agonist commonly used in induced spawning in fish farms (62), and these stimulatory effects were also mimicked by parallel treatment with the native forms of GnRH, namely GnRH2 and GnRH3. These results suggest that GnRH can act directly at the pituitary level to stimulate PRL secretion, gene expression and protein translation, probably via GnRHR_4_ activation in carp lactotrophs. Using functional expression of GnRHR in HEK293 cells, the four GnRHRs in grass carp were shown to have differential selectivity for the two native forms of GnRH, with GnRHR_1_ exhibiting a strong preference for GnRH3, GnRHR_2_ and GnRHR_3_ with higher selectivity for GnRH2, and GnRHR_4_ with similar preference for GnRH2 and GnRH3 (35). Our dose-dependence studies with GnRH2 and GnRH3 in carp pituitary cells revealed that the potency and efficacy for PRL release caused by the two forms of GnRH were quite comparable and the promiscuity of the PRL-releasing effect for the two GnRHs is consistent with the ligand selectivity of GnRHR_4_. Interestingly, the corresponding effects on PRL mRNA expression were found to be more selective for GnRH3 than GnRH2 in terms of the maximal responses and ED_50_ values. These results are intriguing and demonstrate for the first time that the ligand selectivity of GnRHR may vary with the biological readout in the same cell model. The higher sensitivity of GnRH3 for PRL gene expression also raises the possibility that GnRH3 may act as the major signal for PRL regulation by GnRH in carp lactotrophs, which corroborates with the role of GnRH3 as the “hypophysiotropic GnRH” for seasonal breeding in cyprinid species (5). Based on previous studies with domain swapping/deletion of C-terminal tail in GnRHRs from GnRHR-I and -II families (see introduction), it is commonly accepted that GnRHR-I without C-terminal tail is resistant to GnRH desensitization and the opposite is true for GnRHR-II with a C-terminal tail. In grass carp, GnRHR_4_ expressed in lactotrophs is a member of GnRHR-II with a C-terminal tail typical of GPCR (35) and yet the PRL responses in terms of hormone secretion, transcript expression and protein production could still be noted after a 48-hr treatment with sGnRH-A, GnRH2 and GnRH3 at micromolar dose. These findings are at variance with the results expected for GnRHR-II (i.e., with refractoriness/even loss of response after prolonged induction) and suggest that GnRHR_4_ expressed in carp lactotrophs is also resistant to GnRH desensitization similar to GnRHR-I.

In mammals, GnRHR can trigger post-receptor signalling via a single type/multiple types of G proteins in different cells/tissues in a context-dependent manner, e.g., with G_q/11_ coupling in pituitary gonadotrophs, G_q/11_ and G_s_ coupling in hypothalamic neurons and G_i_ coupling in cancer cells (e.g., prostate cancer) (69). Based on sequence mutations/domain swapping in rodent GnRHR expressed in COS-7/GGH3 cell lines, the 2^nd^ and 3^rd^ intracellular loops have been confirmed to be the structural domains in GnRHR for functional coupling with G_s_ and G_q/11_ (70, 71) and activation of these G proteins upon ligand binding in GnRHR can lead to signal transduction via the PLC/IP_3_/PKC, AC/cAMP/PKA, MAPK and Ca^2+^-dependent cascades (24, 25). For LH release induced by GnRH, the PLC/IP_3_/PKC pathway with IP_3_-mediated [Ca^2+^]i release and PKC coupling with MAPK forms a major core of the post-receptor signalling for GnRH at the pituitary level and the AC/cAMP/PKA pathway is not involved (27). In fish models, despite the similarity for the involvement of PLC/IP_3_/PKC pathway and the associated Ca^2+^ responses (27, 63), discrepancy on the role of cAMP in GnRH signalling has been reported, e.g., the cAMP-dependent signalling is absent in the LH-releasing effect of GnRH in goldfish (26) and catfish pituitary cells (72) but cAMP/PKA involvement in gonadotropin regulation by GnRH has been documented in tilapia (63) and more recently in grass carp (35). Although PRL release induced by GnRH mediated by G_s_ and cAMP has been reported in GGH3 cells with stable transfection of rat GnRHR (47), whether the phenomenon can occur in primary culture of pituitary cells has yet to be examined. In our study with carp pituitary cells, AC activation by forskolin or increasing functional level of cAMP by 8Br.cAMP was shown to elevate PRL release and PRL mRNA levels and these stimulatory effects could be mimicked by the cAMP analog specific for PKA (6-Bnz-cAMP) but not Epac (pCPT-O-Me-cAMP), implying that activation of AC/cAMP/PKA pathway can trigger PRL secretion and gene expression in carp lactotrophs. This idea agrees with our findings that the PRL responses caused by forskolin could be blocked by AC inhibition with MDL 12330A or PKA inactivation by H89. For the role of cAMP-dependent pathway in PRL regulation by GnRH, GnRH treatment in carp RPD cells could trigger parallel rises in cAMP production and CREB phosphorylation followed by nuclear translocation of CREB, and these signalling events were mimicked by AC activation with forskolin. In parallel experiments with carp pituitary cells, the stimulatory effects of GnRH on PRL secretion and transcript expression were found to be sensitive to G_s_ blockade by melittin, AC inhibition by MDL 12330A, and PKA inactivation by H89. Consistent with the idea of PKA phosphorylation of CREB followed by its nuclear translocation and recruitment of the co-activator CBP to initiate target gene transcription (73), PRL gene expression induced by GnRH could be negated by inactivating CREB with KG-501 or interfering CREB: CBP interaction by SGC-CBP30. Our findings, as a whole, suggest that PRL release and gene expression caused by GnRH in carp lactotrophs are mediated by G_s_ activation coupled to AC/cAMP/PKA pathway. As a well-conserved CRE site can be located upstream of the TATA box within the 5’promoter of grass carp PRL gene, the PRL mRNA response caused by GnRH probably is the result of PRL gene transcription mediated by CREB/CBP complex acting at the promoter level. Given that (i) chromatin remodelling is involved in GnRH-induced gonadotropin gene expression (74), (ii) CBP can induce histone acetylation via its intrinsic HAT activity/by recruitment of PCAF (75), and (iii) PRL mRNA expression induced by GnRH in carp pituitary cells was also sensitive to the blockade of HAT activity with C646, we do not exclude the possibility that modulation of chromatin packaging via CREB/CBP complex coupled with the cAMP-dependent pathway may play a role in PRL gene transcription induced by GnRH in carp species.

In RPD culture prepared from tilapia pituitaries, PRL secretion could be induced by GnRH with parallel rises in IP_3_ levels and [Ca^2+^]i signals and this PRL-releasing effect was ablated by PLC inactivation, VSCC blockade and inhibition of [Ca^2+^]i mobilization (51). Although the functional role of PKC on PRL secretion was not examined, the results suggest that the PLC/IP_3_ pathway and associated Ca^2+^ responses are involved in PRL regulation by GnRH in fish model. In our study, PRL release and PRL mRNA levels could be up-regulated in carp pituitary cells by the DAG analog DiC8 or PKC activator TPA and these effects could be blocked by inactivating PKC with calphostin C, suggesting that PKC activation by DAG can induce PRL secretion and gene expression in carp lactotrophs. In carp pituitary cells, PRL release induced by GnRH was sensitive to G_q/11_ inhibition with YM254890, PLC blockade by U73122, and PKC repression using calphostin C. Although G_q/11_ inhibition could suppress PRL transcript expression caused by GnRH, similar blockade of PLC and PKC was found to have no effect in this regard. Furthermore, IP_3_ antagonism with xestospongin C or blocking IP_3_ channel for [Ca^2+^]i mobilization with 2-APB did not alter GnRH-induced PRL release and gene expression. These results suggest that G_q/11_ activation induced by GnRH can trigger PRL release in carp lactotrophs via PLC/PKC pathway and the IP_3_ component working downstream of PLC is not involved. Apparently, PRL gene expression caused by GnRH is not mediated by the PLC/IP_3_/PKC pathway, which is consistent with our findings that the PKC-sensitive transcription factors, namely c-Fos and c-Jun, did not show noticeable changes in carp RPD cells with GnRH treatment. In our study, PKC activation by TPA or DiC8 could elevate PRL mRNA levels but similar response on PRL gene expression by GnRH did not involve PKC signalling and yet PKC involvement has been clearly implicated for the PRL-releasing effect of GnRH. In living cells, multiple forms of PKC are expressed and TPA and DAG are known to activate the conventional (PKC_α, β & γ_) and novel PKCs (PKC_δ, ε, η & θ_) but not atypical PKCs (PKC_ζ & ι)_ (76). It is tempting to speculate that a subtype of atypical PKCs may be involved in PRL release but not gene expression induced by GnRH. Of note, G_q/11_ inhibition could block PRL transcript expression caused by GnRH and yet the PLC/IP_3_/PKC pathway coupled to G_q/11_ was not involved, functional mediation by other signalling events working downstream of G_q/11_ (e.g., MAPK) for PRL gene transcription cannot be excluded.

Although [Ca^2+^]i mobilization via IP_3_ is well-documented for the LH-releasing effect of GnRH, voltage-gated [Ca^2+^]e influx caused by functional interplay of a combination of ion channels also contributes to the LH response via the action potentials and Ca^2+^ oscillations induced by GnRH at the gonadotroph level (77). The involvement of [Ca^2+^]i mobilization and voltage-gated [Ca^2+^]e entry is well-conserved in GnRH actions in fish species, e.g., for GnRH-induced LH and GH release in goldfish pituitary cells (26, 27). In our study, VSCC activation by Bay K8644 was effective in triggering a rapid rise in cytosolic Ca^2+^ in carp RPD cells and this effect was blocked by inhibiting L-type VSCC with nicardipine. Bay K8644 also increased PRL release and PRL mRNA level in carp pituitary cells and these stimulatory actions could be nullified by L-type VSCC inactivation using nicardipine or CaM antagonism by calmidazolium. For the signalling events working downstream of CaM, blocking CaMK-II activity by KN62 was found to ablate the PRL-releasing effect of Bay K8644 but not the corresponding response on PRL mRNA, whereas the opposite was true for parallel inhibition of CaN using cytosporin A. These results imply that [Ca^2+^]e entry via L-type VSCC can trigger PRL responses via Ca^2+^/CaM-dependent signalling, with PRL release caused by CaMK-II activation and PRL gene expression mediated by CaN-dependent mechanisms. In carp RPD cells, GnRH was effective in inducing Ca^2+^ signals, which could be negated by nicardipine inhibition of L-type VSCC. Parallel kinetic chase of the spatiotemporal distribution of the Ca^2+^ signals in immuno-identified lactotrophs also revealed that the Ca^2+^ rise caused by GnRH was initiated close to the plasma membrane and spread into the central region of responsive cells, which is consistent with [Ca^2+^]e entry via VSCC at the membrane level. During the process, the highest level of Ca^2+^ signals was located in the subplasmalemmal space under the plasma membrane, which is well-documented to be the region with the readily releasable pool of secretory vesicles with proper priming/docking for Ca^2+^-sensitive exocytosis in endocrine cells (78). In carp pituitary cells, PRL secretion and gene expression caused by GnRH were also sensitive to VSCC blockade with nicardipine and CaM antagonism with calmidazolium. For the downstream events coupled to Ca^2+^/CaM signalling, the PRL-releasing effect of GnRH but not the corresponding action on PRL transcript could be obliterated by inactivating CaMK-II with KN62. However, the elevation in PRL mRNA levels but not PRL release caused by GnRH was reduced/nullified by blocking CaN with cyclosporin A, interfering CaN:NFAT interaction by INCA-6, or inhibiting nuclear translocation of NFAT using 11R-VIVIT. These results are also consistent with our finding that GnRH treatment was effective in reducing NFAT_2_ phosphorylation in carp RPD cells. Since (i) CaMK-II activation by Ca^2+^/CaM complex can phosphorylate exocytosis regulators (e.g., syntaxin and synapsin) for priming/docking of secretory vesicles at membrane level prior to their exocytosis (79), (ii) CaN is a serine/threonine phosphatase and its enzyme activity can be up-regulated by Ca^2+^/CaM signalling to trigger gene transcription via NFAT dephosphorylation (80), and (iii) three NFAT binding sites with the proximal one overlapping with the CRE site upstream of the TATA box can be located in the 5’ promoter of grass carp PRL gene, our findings may imply that Ca^2+^/CaM signalling caused by [Ca^2+^]e entry via L-type VSCC is involved in PRL regulation by GnRH. Apparently, the PRL-releasing effect is mediated by Ca^2+^/CaM activation of CaMK-II and the corresponding response for PRL transcript is caused by PRL gene transcription via parallel activation of CaN/NFAT_2_. In grass carp, differential involvement of CaMK-II and CaN/NFAT in negative feedback of GH by IGF-I has also been reported (58). In this case, IGF-I could inhibit GH gene transcription in carp pituitary cells by elevating CaM expression with parallel rises in CaN/NFAT but not CaMK-II signalling. In carp RPD cells, the Ca^2+^ responses for GnRH could be totally negated by nicardipine and this observation suggest that the Ca^2+^ signals in lactotrophs caused by GnRHR activation are mainly of extracellular origin. Together with our findings that IP_3_ antagonism/IP_3_ channel blockade did not alter the PRL responses by GnRH, it would be logical to assume that IP_3_-induced [Ca^2+^]i mobilization is not a major component in the signal transduction for PRL regulation by GnRH. This idea, however, is at variance with the reports on LH release induced by GnRH, in which the PLC/IP_3_ pathway coupled to [Ca^2+^]i mobilization forms a major core of the post-receptor signalling in gonadotrophs (25, 27).

In grass carp, the proximal NFAT binding site was found to overlap with the CRE site identified in the 5’promoter of PRL gene, which raises the possibility that a functional crosstalk between cAMP- and Ca^2+^-dependent pathways may be involved in PRL regulation in carp species. In fish models, functional coupling of cAMP/PKA pathway with Ca^2+^-dependent signalling has been reported for pituitary hormone secretion/gene expression. For examples, cAMP signalling coupled with Ca^2+^ entry via VSCC was shown to mediate GH secretion induced by dopamine via D1 receptor activation in goldfish pituitary cells (81, 82). In carp pituitary cells, PACAP via PAC1 receptor activation can act as a potent stimulator for GH (57), PRL (55) and SLα gene expression (83), and these stimulatory effects also involve cAMP/PKA-mediated Ca^2+^ entry via VSCC and Ca^2+^/CaM activation of CaMK-II. In our previous study in grass carp, CaM gene was cloned, and its promoter activity was shown to be under the control of cAMP signalling (84). Furthermore, CaM expression in carp pituitary cells could be up-regulated by PACAP in a cAMP/PKA-dependent manner (55) and contributes to PACAP-induced PRL promoter activition (85) and transcript expression (55). Apparently, the crosstalk of cAMP pathway with Ca^2+^-dependent signalling can occur at two levels in the carp pituitary, with (i) VSCC activation to allow [Ca^2+^]e entry to jack up cytosolic Ca^2+^ and (ii) increasing CaM expression to enhance the sensitivity to Ca^2+^ signals. In our study with carp RPD cells, a rise in Ca^2+^ signal was noted with AC activation by forskolin and this Ca^2+^ response could be blocked by inhibiting L-type VSCC with nicardipine or PKA inactivation using H89, suggesting that L-type VSCC can be activated by cAMP signal in lactotrophs via PKA activation. This idea also corroborates with the reports that the ion channel activity of VSCC can be altered by protein phosphorylation (86), e.g., enhancement of inward Ca^2+^ current/channel opening probability is well-documented for PKA phosphorylation of Ca_V_1.4 (87) and Ca_V_1.2a,b subtypes of L-type VSCC (88). In carp pituitary cells, VSCC inhibition with nicardipine and CaM antagonism with calmidazolium were both effective in blocking PRL release and PRL gene expression caused by forskolin. Furthermore, the PRL-releasing effect of forskolin but not the parallel stimulation on PRL mRNA could be ablated by CaMK-II inhibition with KN62 while the opposite was true with CaN inactivation by cyclosporin A. Similar to GnRH, forskolin also induced NFAT_2_ dephosphorylation in carp RPD cells. In the same cell model, CaM expression could be up-regulated by GnRH and the effect was mimicked by AC activation with forskolin, increasing cAMP level by 8Br.cAMP, and PKA activation using 6-Bnz-cAMP. Consistent with CaM expression at protein level, GnRH treatment could also increase CaM mRNA level in RPD cells, and the effect was sensitive to G_s_ inhibition using melittin, AC repression by MDL 12330A, PKA blockade with H89, CREB inactivation by KG-501, and blocking CREB:CBP interaction by SGC-CBP30. These findings, as a whole, imply that (i) the Ca^2+^/CaM-dependent signalling with involvement of CaMK-II in PRL secretion and CaN/NFAT in PRL gene expression are working downstream of the cAMP/PKA pathway coupled with G_s_ signalling, and (ii) the crosstalk between cAMP- and Ca^2+^-dependent cascades induced by GnRH in carp lactotrophs probably is mediated by [Ca^2+^]e entry via PKA activation of L-type VSCC and CaM expression coupled with AC/cAMP/PKA signalling via CREB/CBP-dependent gene transcription.

**Figure 13:**
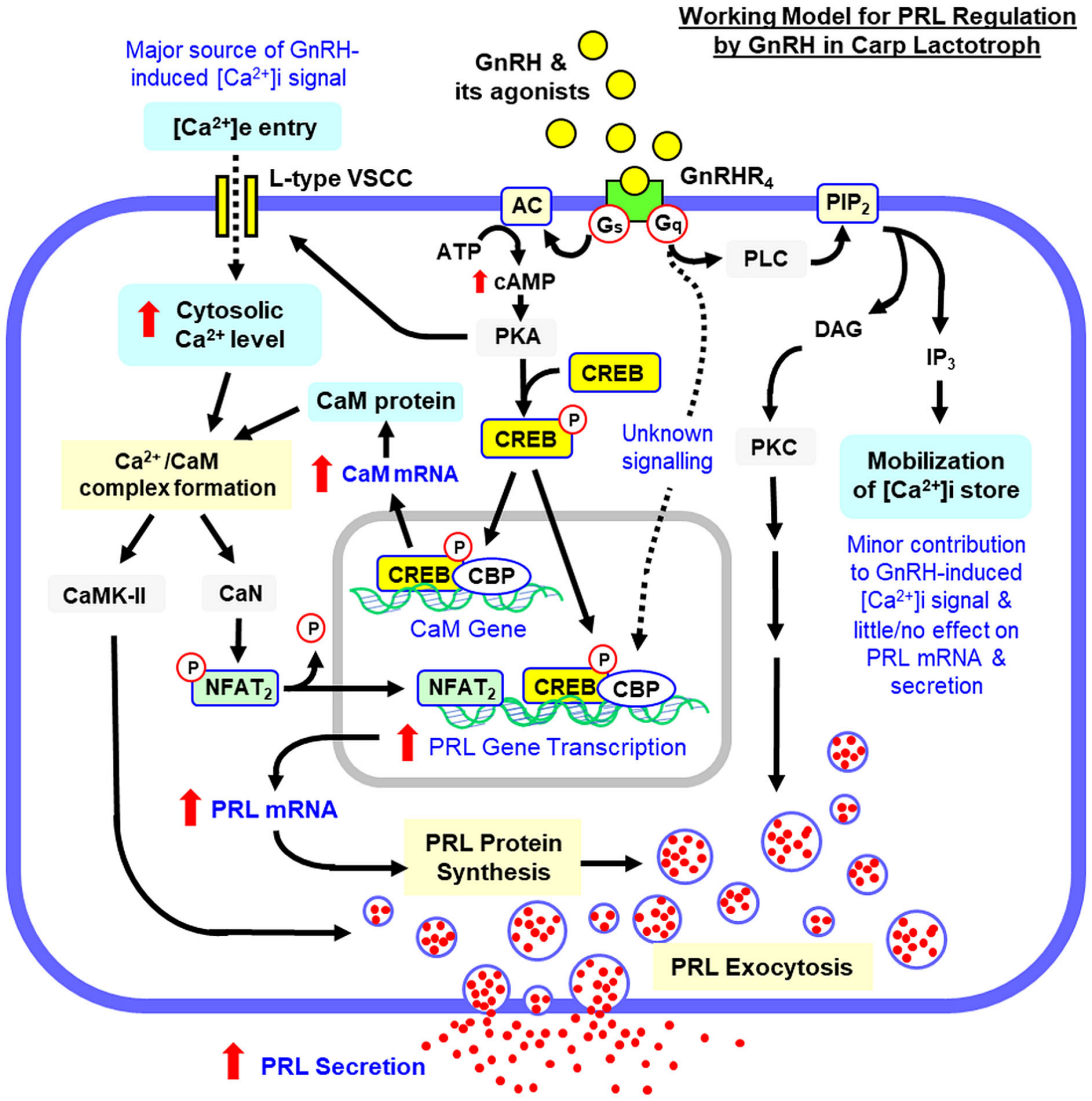
Working model for signal transduction involved in GnRH-induced PRL secretion and gene expression in carp lactotrophs. In this model, native GnRHs (GnRH2/3) or its functional agonists (e.g., sGnRH-A) bind with GnRHR_4_ expressed in carp lactotrophs and induce post-receptor signalling via G_s_ and G_q/11_ coupling. G_s_ coupling with AC can increase cAMP production to trigger PKA activation with rapid phosphorylation and nuclear translocation of CREB. Through binding with PRL promoter, CREB recruits the co-activator CBP to initiate PRL gene transcription and the subsequent rise in PRL transcript expression presumably can contribute to PRL exocytosis by increasing PRL synthesis. PKA activation can also induce [Ca^2+^]e entry via activation of L-type VSCC. The subsequent rise in Ca^2+^ signals in the cytoplasm through binding with the Ca^2+^ sensor CaM can induce PRL secretion via CaMK-II activation and elevate PRL transcript expression via CaN-induced dephosphorylation of NFAT_2_, which is assumed to work with CREB/CBP at the promoter level to trigger PRL gene transcription. CREB/CBP coupled to cAMP/PKA pathway can also induce CaM gene expression with subsequent rise in CaM signal to enhance Ca^2+^/CaM-dependent activation of CaMK-II and CaN/NFAT signalling. Parallel to the post-receptor signalling via G_s_, GnRHR_4_ activation can also trigger PRL exocytosis via G_q/11_ coupling to the PLC/DAG/PKC pathway. Apparently, [Ca^2+^]i mobilization by IP_3_ working downstream of PLC is not involved in PRL secretion and gene expression induced by GnRH.

In summary, using grass carp pituitary cells as a model, we have demonstrated for the first time that the cAMP/PKA-, PLC/PKC- and Ca^2+^/CaM-dependent signalling are differentially involved in PRL secretion and gene expression induced by GnRH at the pituitary level. Based on the results obtained, a working model has been proposed for the signal transduction mediating PRL regulation by GnRH in carp lactotrophs (**Figure 13**). In this model, GnRHR_4_ activation in lactotrophs by GnRH2/3 or their agonist sGnRH-A can initiate post-receptor signalling via G_s_ and G_q/11_ coupling. The AC/cAMP/PKA pathway and [Ca^2+^]e entry via L-type VSCC with subsequent Ca^2+^/CaM-activation of CaMK-II and CaN are coupled to G_s_ activation while the PLC/IP_3_/PKC pathway with downstream PKC-dependent signalling and [Ca^2+^]i mobilization by IP_3_ are linked with G_q/11_ activation. Interestingly, different components of these signalling events are differentially involved in PRL secretion and gene expression by GnRH, with (i) the AC/cAMP/PKA, Ca^2+^/CaM/CaMK-II and PLC/DAG/PKC signalling responsible for the PRL-releasing effect, and (ii) PKA-induced CREB phosphorylation for nuclear entry and CBP recruitment and Ca^2+^/CaM-mediated NFAT_2_ dephosphorylation by CaN to trigger PRL gene transcription with subsequent rise in PRL mRNA and protein synthesis. In carp lactotrophs, the Ca^2+^-dependent signalling via CaMK-II (for PRL release) and CaN activation (for PRL gene expression) is working downstream of the AC/cAMP/PKA pathway probably through (i) VSCC activation by PKA to induce [Ca^2+^]e entry at membrane level and (ii) CaM up-regulation via transcriptional activation by CREB/CBP coupled to cAMP/PKA signalling. Although the other aspects of post-receptor signalling (e.g., MAPK) have not been touched in PRL regulation in our cell model, our study has provided novel information on the complexity of signal transduction involved in PRL release and gene expression by GnRH in a carp species. Given that our study focused on the pituitary actions of GnRH using primary culture of pituitary cells, further investigations using an *in vivo* approach (e.g., for GnRH interactions with other PRL regulators of central origin/from the periphery or GnRHR knockout in lactotrophs using CRISPR/Cas9) are clearly warranted to establish a more holistic picture for the physiological role of GnRH in PRL regulation. It is also worth mentioning that the pituitary cells used in our study were prepared from pre-pubertal fish, it will be of interest to know if the post-receptor signalling for PRL regulation by GnRH can be modified/“fine-tuned” with sexual maturation/with different stages of the seasonal reproductive cycle in adult fish as in the case of gonadotropin secretion/gene expression.

## Data availability statement

The original contributions presented in the study are included in the article/**Supplementary Material**. Further inquiries can be directed to the corresponding author.

## Ethics statement

The animal study was approved by The Committee on the Use of Live Animal for Teaching and Research, the University of Hong Kong, Hong Kong. The study was conducted in accordance with the local legislation and institutional requirements.

## Author contributions

WL: Conceptualization, Data curation, Investigation, Methodology, Writing – review & editing, Formal analysis. CY: Data curation, Formal analysis, Investigation, Methodology, Software, Writing – review & editing. MH: Data curation, Formal analysis, Investigation, Methodology, Validation, Writing – review & editing. WK: Data curation, Formal analysis, Investigation, Methodology, Software, Validation, Visualization, Writing – review & editing. CC: Conceptualization, Funding acquisition, Project administration, Supervision, Writing – original draft. YC: Project administration, Supervision, Visualization, Writing – review & editing. AW: Conceptualization, Data curation, Formal analysis, Funding acquisition, Methodology, Project administration, Resources, Supervision, Writing – original draft, Writing – review & editing.

## In memoriam

This paper is dedicated to Prof. Christopher H. K. Cheng (Chinese University of Hong Kong, HK), a collaborator and long-term friend who passed away before the project was completed. Chris’s passion for comparative endocrinology will always be missed and the students he trained throughout the years (many of them are now prominent scientists in Hong Kong and mainland China) will continue to make notable contributions to our research community for years to come.
