# Supplementary material for: Differential involvement of cAMP/PKA-, PLC/PKC- and Ca2+/calmodulin-dependent pathways in GnRH-induced prolactin secretion and gene expression in grass carp pituitary cells
Source: Front Endocrinol (Lausanne). 2024 Jun 4;15:1399274. doi: 10.3389/fendo.2024.1399274 (PMC11183098; doi:10.3389/fendo.2024.1399274)
Supplement: Supplementary file 1 [file DataSheet_1.pdf]

Supplemental Table S1 & S2

Supplemental Table S1 Primers and PCR conditions used in real-time PCR for PRL and CaM mRNA

Gene Target / GenBank Accession No.<br>Sequences of Forward (F) & Reverse Primers (R)	Real-time PCR condition					Product size & T_m value
	Denaturing	Annealing	Extension	Detection	Cycle	
Grass Carp PRL / EU074210<br>F: 5'-CTCAGCACCTCTCTCACCAATGACC-3'<br>R: 5'-GCGGAAGCAGGACAACAGAAAATG-3'	94 °C<br>30 sec	60 °C<br>30 sec	72 °C<br>30 sec	90 °C<br>20 sec	x 35	400 bp<br>T_m = 93.6 °C
Grass Carp CaM / AY627883<br>F: 5'-AGTCTGAGCCTCCCATTTACAC-3'<br>R: 5'-CAGCCTTGATGCGTTCTT-3'	94 °C<br>30 sec	69 °C<br>30 sec	72 °C<br>30 sec	86 °C<br>20 sec	x 35	181 bp<br>T_m = 89.8 °C
Grass Carp 18S RNA / HQ615531<br>F: 5'-AGCAACTTTAGTATACGCTATTGGAG-3'<br>R: 5'-CCTGAGAAACGGCTACCACATCC-3'	94 °C<br>30 sec	56 °C<br>30 sec	72 °C<br>30 sec	87 °C<br>20 sec	x 35	285 bp<br>T_m = 91.2 °C

Supplemental Table S2 Primers and PCR conditions used in RT-PCR for tissue expression of $GnRHR_{1\text{-}4}$

Gene Target / GenBank Accession No.<br>Sequences of Forward (F) & Reverse Primers (R)	PCR condition				Product size
	Denaturing	Annealing	Extension	Cycle	
Grass Carp $GnRHR_1$ / OP482096<br>F: 5'-CACCGACATTCACCGTTGCT-3'<br>R: 5'-CAGACTCACCACCACCAGGAT-3'	94 °C<br>30 sec	60 °C<br>30 sec	72 °C<br>30 sec	x 40	317 bp
Grass Carp $GnRHR_2$ / OP482097<br>F: 5'-CGTGCTTTACTCAGCCAACC-3'<br>R: 5'-CCTCCTCGCTTTGTTGATAG-3'	94 °C<br>30 sec	59 °C<br>30 sec	72 °C<br>70 sec	x 40	280 bp
Grass Carp $GnRHR_3$ / OP433498<br>F: 5'-GCAGGAGACGGCGTACAACAT-3'<br>R: 5'-CCAGCAGACCACGAACGACAT-3'	94 °C<br>30 sec	62 °C<br>30 sec	72 °C<br>70 sec	x 40	226 bp
Grass Carp $GnRHR_4$ / OP433499<br>F: 5'-CATCATGATCTGCTGCTACAC-3'<br>R: 5'-CTCAGGAAGGAACCAGTACC-3'	94 °C<br>30 sec	60 °C<br>30 sec	72 °C<br>70 sec	x 40	219 bp
Grass Carp β-actin / AB039726.2<br>F: 5'-CTGGTATTCGTGATGGACTCT-3'<br>R: 5'-AGCTCATAGCTCTTCTCCAG-3'	94 °C<br>30 sec	56 °C<br>30 sec	72 °C<br>30 sec	x 35	280 bp

* For RT-PCR based on carp lactotrophs captured by LCM, the cycle no for PCR amplification was increased to x 55 cycles.
