# Supplementary material for: Differential involvement of cAMP/PKA-, PLC/PKC- and Ca2+/calmodulin-dependent pathways in GnRH-induced prolactin secretion and gene expression in grass carp pituitary cells
Source: Front Endocrinol (Lausanne). 2024 Jun 4;15:1399274. doi: 10.3389/fendo.2024.1399274 (PMC11183098; doi:10.3389/fendo.2024.1399274)
Supplement: Supplementary file 2 [file DataSheet_2.pdf]

Supplemental Fig.S1

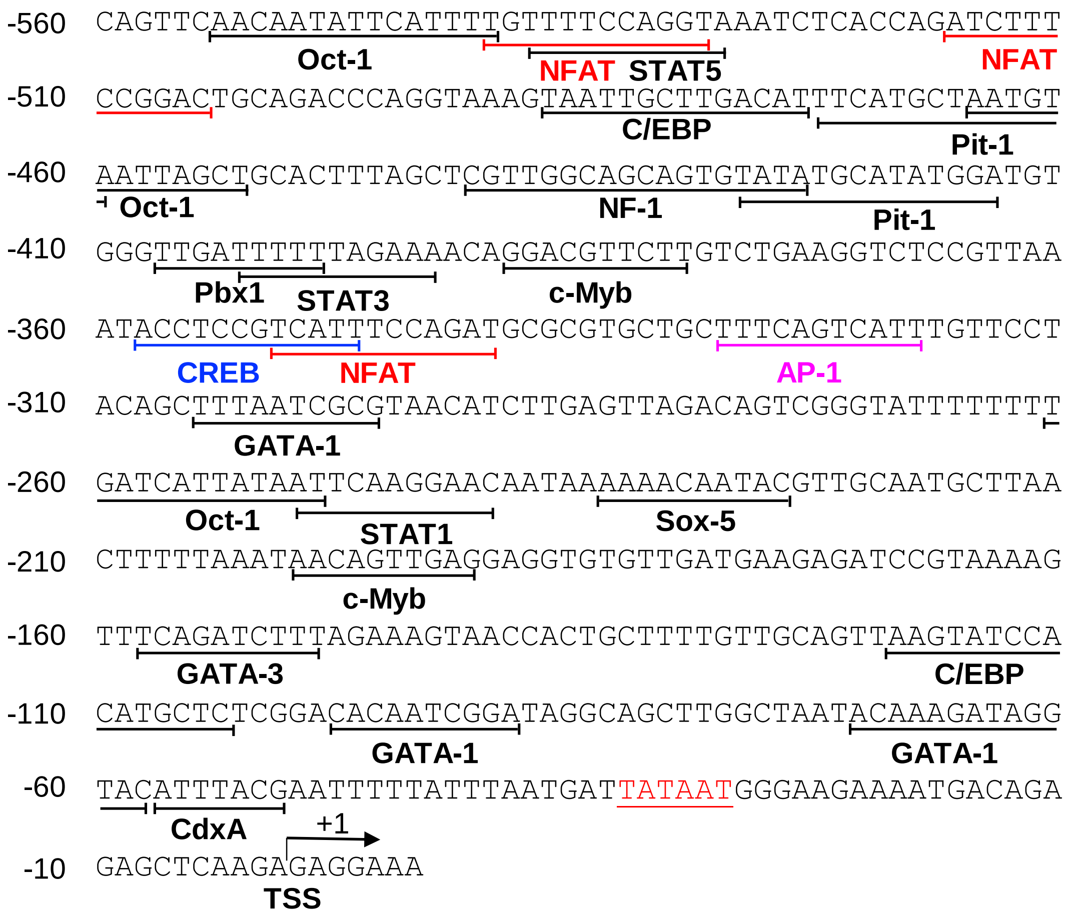

<u>Supplemental Fig.S1</u> Promoter analysis of grass carp PRL gene (Gene ID: 127509752) for binding sites for transcription factors (TF). The proximal promoter (up to position -560) of carp PRL gene was downloaded from NCBI database and subjected to TF site search using TransFAC® 2.0 (https://genexplain.com/transfac/). The transcription start site (TSS) was taken as "+1" to serve as a reference point for numbering of promoter sequence while the TF binding sites identified were delineated by horizontal bars under the respective sequences. Within the proximal region of PRL promoter, one CRE site (with core sequence "CGTCA"; underlined with blue line), one AP-1 site (with core sequence "TGAGTCA"; underlined with pink line) and three NFAT binding sites (with core sequence "GGAAA" in reverse orientation; underlined with red lines) were located upstream of the TATA box (labelled in red). Of note, the proximal NFAT binding site was found to overlap with the CRE site in the PRL promoter.
