# Supplementary material for: Differential involvement of cAMP/PKA-, PLC/PKC- and Ca2+/calmodulin-dependent pathways in GnRH-induced prolactin secretion and gene expression in grass carp pituitary cells
Source: Front Endocrinol (Lausanne). 2024 Jun 4;15:1399274. doi: 10.3389/fendo.2024.1399274 (PMC11183098; doi:10.3389/fendo.2024.1399274)
Supplement: Supplementary file 3 [file DataSheet_3.pdf]

Supplemental Fig.S2

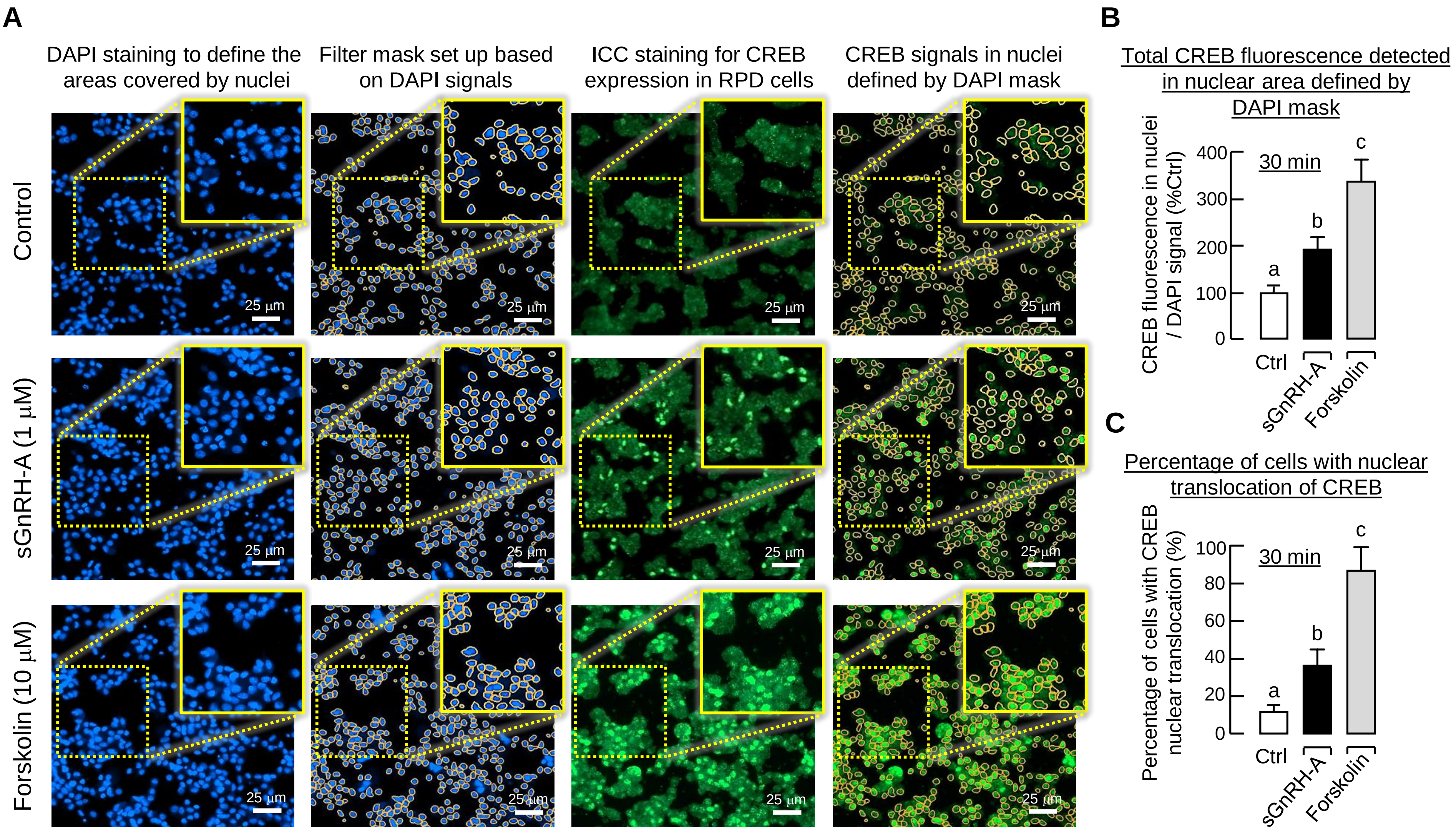

Supplemental Fig.S2 Quantitative analysis of CREB nuclear translocation. RPD cells were incubated with sGnRH-A (1 µM) or forskolin (10 µM) for 30 min followed by fluorescence ICC staining with antibody for total CREB (1:1000) and nuclear staining with DAPI. (A) Fluorescence images were captured using a Cytation-1 Multimodel Reader with CF488A signals for CREB and DAPI signals for nuclear staining. Using Gen 5 software, a filter mask was constructed for the nuclei of RPD cells using the DAPI signals and used for quantitation of nuclear signals of CREB and cell counting of RPD cells with notable signals of CREB in the nucleus. Based on image analysis with the "DAPI mask", (B) the total fluorescence of CREB signals detected in the nuclei of RPD cells and (C) percentage of RPD cells with nuclear translocation of CREB were found to be significantly enhanced by sGnRH-A and forskolin treatment, respectively.
